# Exploring the motivation and structural barriers of women’s sports consumption from the perspective of the “She-Economy”

**DOI:** 10.1371/journal.pone.0359201

**Published:** 2026-10-21

**Authors:** Yixuan Shi, Tao Yang, Kaidi Zhang, Cuixia Yi

**Affiliations:** 1 School of Sports Economics and Management, Xi’an Physical Education University, Xi’an, China; 2 Guangxi Normal University, Guilin, China; 3 Shaanxi Normal University, Xi’an, China; ESECS-Polytechnique of Leiria / Research Center in Sports Sciences, Health Sciences and Human Development, CIDESD, PORTUGAL

## Abstract

Against the backdrop of consumption upgrading and growing gender consciousness, the “She-Economy” has emerged as an important socio-economic phenomenon driving market transformation and consumption restructuring. Women’s participation in sports consumption has continued to increase, making women a significant consumer group within the sports industry. However, women’s sports consumption is a contradictory field in which empowerment, identity formation, market discipline, gender norms, institutional neglect, and structural inequality coexist. Therefore, this study adopted a descriptive phenomenological design and employed literature review and semi-structured in-depth interviews with nine Chinese women who had sports consumption experience. Colaizzi’s seven-step method was used to conduct thematic analysis of the interview data in order to explore the motivations and structural barriers underlying women’s sports consumption within the context of the “She-Economy”. The findings reveal that women’s sports consumption is driven by multiple motivations, including health management, social interaction, self-realization, and market-driven incentives. Sports consumption not only reflects women’s pursuit of physical health and emotional well-being, but also demonstrates the growing awakening of female subjectivity and lifestyle autonomy. However, women continue to face structural constraints in the process of sports consumption, including time pressure, economic costs, gender-role expectations, male-dominated sports spaces, and insufficient institutional support. These findings reflect a paradoxical coexistence between strengthened consumption motivation and restricted behavioral practice. The findings extend current understanding of women’s sports consumption under the “She-Economy” by showing that women’s sports consumption is a socially negotiated practice in which individual agency and structural constraints continuously interact. The study also provides exploratory, context-specific insights into women-centered sports products and services, supportive socio-cultural environments, and more gender-responsive sports services, offering qualitative evidence for future research and practice.

## Introduction

The term ‘She-Economy’ (female-driven economy) has been widely discussed in China to describe the growing economic influence of female consumers. In recent years, with the advancement of social and economic development and the growing awareness of gender equality, the She-Economy has emerged as a new economic phenomenon and has become an important force driving structural transformation in the consumer market. The expansion of consumerism and materialism has further stimulated women’s investment in their bodies, with the expectation of receiving visible and tangible returns [1]. Unlike traditional female consumption patterns that primarily emphasized family-oriented and functional consumption, the “She-Economy” increasingly highlights self-development, emotional experience, and self-expression. Previous studies have suggested that women’s consumption behavior is gradually shifting from satisfying family needs toward fulfilling individual developmental and identity-related needs [2]. Women’s sports participation and sports consumption have become important drivers of growth in the global sports industry [3]. Existing international studies have shown increasing female participation in fitness, outdoor sports, and digital fitness consumption, with women emerging as an important consumer group in the global sports market [2,4].

China has actively promoted sports consumption and mass fitness as part of broader consumption upgrading and public health strategies [5]. Among various consumption fields, sports consumption has become a key component of lifestyle upgrading and leisure culture transformation, with multiple functions including health improvement, social interaction, and self-fulfillment. In practice, however, a contradiction has emerged: while women, driven by the wave of the She-Economy, show a strong willingness to engage in sports-related consumption—such as fitness courses, sportswear, and sports event participation—this intention has not been fully converted into stable consumption behavior. Although the emergence of the She-Economy reflects women’s increasing economic participation and market visibility, it should not be understood simply as evidence of women’s liberation or autonomous consumption. In this sense, the She-Economy represents not only an expanding consumer market but also a contradictory social formation in which women negotiate personal aspirations, identity construction, market expectations, and gendered norms.

Existing studies have predominantly focused on market trends and quantitative analyses [6], while paying insufficient attention to women’s lived experiences, subjective motivations, and perceived barriers in everyday sports consumption practices. To address these gaps, this study employs gender theory and consumption sociology theory as complementary analytical frameworks to explore how women’s sports consumption motivations and structural barriers are socially and culturally constructed. Against this backdrop, the present study seeks to answer the following questions: Why does women’s sports consumption under the She-Economy demonstrate the coexistence of strong intention and limited action? What motivations drive women’s participation in sports consumption? And what structural barriers constrain the realization of these motivations in practice, and in what ways do women enact their agency amidst these structural constraints? By addressing these questions, this study aims to reveal the underlying social logic of women’s sports consumption behavior and provide insights for promoting more gender-inclusive development within the sports industry in the new era.

## 1. Overview of the “She-Economy” and its influence on women’s sports consumption

### 1.1 The connotation and rise of the “She-Economy”

The term “She-Economy”, also known as the female economy, was first introduced by economist Shi Qingqi at the Ninth National Women’s Congress in China. It refers to a distinct economic phenomenon and market sphere shaped around women’s growing participation in consumption, financial management, and lifestyle decision-making. Shi emphasized that an increasing number of enterprises have begun to identify and design products from a female perspective, recognizing women as a core consumer group. Economic scholars further suggest that women’s financial independence, strong purchasing power, and rising consumption demand signal the formation of a new engine for economic growth. The essence of the She-Economy lies in the emergence of modern women—especially those who are well educated and financially autonomous—as a dynamic force driving consumption upgrading and industrial transformation. The rise of this phenomenon is not coincidental but rather the result of intersecting social, economic, and cultural transformations. Socially, the expansion of higher education and the advancement of gender equality movements have significantly improved women’s socioeconomic status, granting them more disposable income and decision-making authority. Economically, the trend toward smaller family structures and the postponement of marriage and childbirth have fostered the development of the “single economy” and the “self-pleasing economy,” both of which further stimulate women’s consumption potential. Culturally, the awakening of women’s self-awareness has shifted consumption priorities from meeting basic material needs toward pursuing emotional satisfaction, social recognition, and self-fulfillment. In this evolving context, women are no longer passive consumers but active definers and leaders of market trends, reshaping diverse industries—from beauty and apparel to tourism, fitness, and beyond—through their distinct consumption preferences and values.

### 1.2 The landscape and characteristics of the women’s sports consumption market

Under the sweeping influence of the She-Economy, the sports consumption market is undergoing a profound transformation driven by women’s participation—often described as a “female revolution.” Women’s sports consumption refers to the purchasing behaviors of women related to participation in sports activities, spectating at sporting events, or obtaining sports-related products and services. It generally includes product-based consumption, participatory consumption, spectator consumption, and media-based consumption. At present, China’s female sports consumption market is characterized by rapid expansion in scale and continuous diversification across sub-sectors. On the demand side, the rise of “female power” (she-power) has significantly enhanced the commercial value of women’s sports participation and related industries. The behavioral patterns of women’s sports consumption reflect three core features that align closely with the essence of the She-Economy: (1) Emotional and self-pleasing orientation—consumption is not only functional but also emotional, aimed at achieving physical and mental health, stress relief, and psychological satisfaction; (2) Community-based and identity-driven participation—women seek social connection and a sense of belonging through running clubs, yoga groups, and online fitness communities; (3) Diverse and personalized demand—women place greater emphasis on product aesthetics, technological performance, cultural meaning, and brand storytelling, demonstrate a willingness to pay for high‑quality experiences and aesthetic value.

## 2. Research design

### 2.1 Study design and participants

This study adopted a descriptive phenomenological design to explore women’s motivations and structural barriers in sports consumption within the context of the “She-Economy.” The choice of phenomenology aligns with the study’s aim of capturing participants’ lived experiences and the meanings constructed through their consumption practices. The research process integrated three methodological components—literature review, semi-structured in-depth interviews, and Colaizzi’s seven-step analysis method—forming a coherent and mutually reinforcing analytical framework.

The literature review served as the theoretical foundation for this study. Through systematic retrieval and analysis of Chinese and international academic works related to the She-Economy, gender studies, sports consumption, and consumption sociology, existing conceptual frameworks and research gaps were identified. Insights from the literature informed the development of the semi-structured interview guide.

Data were analyzed using Colaizzi’s seven-step phenomenological method. NVivo 12 software was used to assist with data management. This rigorous analytic process ensured transparency, reliability, and reproducibility of the findings.

Participants were recruited through purposive and snowball sampling based on the following inclusion criteria: (1) female; (2) aged 18 and above; (3) with at least one form of sports consumption experience within the past 12 months; and (4) willing to participate in a recorded interview. Exclusion criteria included: (1) individuals unable to communicate effectively due to health conditions, and (2) professional athletes or sports industry practitioners to avoid role bias. To reduce potential homogeneity bias associated with snowball sampling, participants were recruited from diverse age groups, occupational backgrounds, marital statuses, and exercise habits in order to improve sample diversity. A total of nine women aged 23–50 were enrolled, representing diverse occupations, marital statuses, and sports participation backgrounds (Table 1). Three participants representing different demographic backgrounds were invited for member checking due to their availability and willingness to participate. Their feedback confirmed that the interpretations accurately reflected their experiences, and no substantial modifications were required.

**Table 1:** Basic information of interview participants.

AD	Age	Level of the city of residence	Occupation	Education	Average Monthly Income (CNY)	Sports Experience	Marital Status
F1	50	Tier 4	Housewife	High school	Unknown	Badminton	Married with children
F2	27	Tier 3	Social media practitioner	Master’s degree	Unknown	Surfing, fitness, swimming	Married without children
F3	47	Tier 2	Public Institution Employee	Bachelor’s degree	7,500	Indoor yoga, aerobics	Married with children
F4	23	New Tier 1	student	Master’s degree	4,500	Swimming, Pamela online workouts	Unmarried, no children
F5	26	New Tier 1	Master’s student	Master’s degree	5,000	Fitness	Unmarried, no children
F6	27	New Tier 1	Beverage barista	Bachelor’s degree	3,800	Badminton, jogging	Unmarried, no children
F7	27	New Tier 1	Master’s student	Master’s degree	4,000	Marathon	Unmarried, no children
F8	30	New Tier 1	University staff	Doctoral degree	9,000	Fitness	Married without children
F9	24	Tier 3	Private education instructor	Bachelor’s degree	3,200	Badminton	Unmarried, no children

One-on-one semi-structured interviews were conducted either face-to-face or online, depending on participant preference. Interview topics included motivations, consumption experiences, interpersonal influences, and perceived structural constraints. The semi-structured interview guide was developed based on previous literature on women’s sports participation, sports consumption, gender norms, and the She-Economy. The questions were reviewed by two researchers with expertise in sports sociology and qualitative methodology. Each interview lasted 30–70 minutes. All interviews were conducted in Chinese, the participants’ native language, to ensure that participants could accurately express their experiences and emotions. With participants’ permission, all interviews were audio-recorded and transcribed verbatim by the researchers shortly after each interview. The transcription process aimed to preserve participants’ original expressions, contextual meanings, and emotional descriptions. The researchers repeatedly compared the transcripts with the original audio recordings to verify accuracy and correct any omissions or misunderstandings. During transcription, all potentially identifying information was removed or replaced with anonymous participant codes (e.g., F1–F9) to protect confidentiality. Monthly income information was unavailable for several participants because some declined to disclose their earnings. Since the original data were collected in Chinese, quotations used in the manuscript were translated into English by the authors, with attention paid to preserving the original meanings. To minimize potential meaning distortion, the translated quotations were repeatedly compared with the original Chinese transcripts, and discrepancies were discussed among the researchers until consensus was reached. Two researchers independently coded all transcripts and resolved discrepancies through discussion.

Although the sample consisted of only nine participants, the determination of thematic saturation was not based solely on the number of interviews, but on the richness, repetition, and conceptual completeness of the emerging data. During the analytic process, each interview was continuously compared with previous transcripts, and new codes, formulated meanings, and thematic dimensions were monitored throughout the coding process. After the eighth interview, the newly identified statements primarily reinforced existing categories related to motivational drivers and structural barriers rather than generating substantively new concepts. The ninth interview further confirmed previously established themes, including time constraints, economic considerations, gender-role expectations, and negotiation strategies adopted by women to maintain sports participation. Moreover, although participants differed in age, occupation, marital status, and sports backgrounds, the recurrence of similar experiential patterns across these diverse social positions suggested that the core structures of the phenomenon had been sufficiently captured. Therefore, saturation was considered achieved when additional interviews no longer contributed new thematic insights relevant to the research questions. Given the phenomenological nature of this study and the relatively small sample size, the findings are intended to provide in-depth insights into participants’ lived experiences rather than statistical generalization to the broader population of women in China.

### 2.2 Data collection

The interview questions, serving as the primary data collection instrument, were developed based on a review of relevant higher-level literature related to the research topic, forming the initial outline for the interviews. It is important to note that as the process of “data collection–analysis” progressed, the actual interview questions were adjusted and refined according to emerging findings and specific contexts. The data collection was conducted between August and November 2025, during which nine female sports enthusiasts with prior sports consumption experiences were interviewed. In total, approximately 10,000 words of transcribed interview data and nine corresponding audio recordings were obtained as reference materials for analysis and verification.

### 2.3 Ethics statement

This study involved non-interventional, minimal-risk qualitative interviews and did not involve medical procedures, sensitive personal information, or vulnerable populations. According to the guidelines of the Academic Affairs Office of Xi’an Physical Education University—which serves as the institution’s ethics oversight body for non-medical human-subject research—such studies are exempt from formal Institutional Review Board (IRB) review. Accordingly, the study received an official certification of exemption from ethical review.

Prior to participation, all interviewees were informed of the study purpose, procedures, confidentiality protections, and their right to withdraw at any time. Both written informed consent and audio-recorded verbal consent were obtained from all participants before each interview. No minors were included in this study. All data were anonymized during transcription and analysis, and no identifiable personal information was retained. The study adhered to the principles of the Declaration of Helsinki.

### 2.4 Researcher reflexivity and positionality

Given that this study explored women’s experiences related to gender, body image, family responsibilities, and consumption practices, researcher reflexivity was considered essential throughout the research process. The researchers acknowledge that their academic training in sports sociology and gender studies, as well as their personal perspectives on women’s participation in sports, could influence the interpretation of participants’ narratives. Therefore, the researchers maintained continuous reflexive awareness throughout data collection and analysis, carefully distinguishing participants’ expressed meanings from researchers’ theoretical assumptions. Regular discussions among the research team were conducted to examine potential interpretive bias and reach consensus during coding and theme development. Rather than seeking statistical agreement, the researchers emphasized interpretive consistency through continuous discussion, reflexive comparison, and consensus-building, which is consistent with the interpretivist orientation of descriptive phenomenological research. This reflexive approach is consistent with the interpretivist perspective emphasized by Rind (2021) [7], who argues that researchers’ positionality and subjective experiences should be critically acknowledged rather than completely eliminated in qualitative inquiry.

## 3. Theoretical framework

### 3.1 Consumption sociology theory: Revealing the cultural connotations of sports consumption

Consumption, as a form of social interaction that connects the economy and culture, serves as the intersection of economic, cultural, and social life, shaping a space where capital and everyday practices are intertwined. From a sociological perspective, consumption is not merely an expression of economic efficiency but a comprehensive process through which individuals engage in cultural construction, spatial mobility, social classification, and the reproduction of social relations. According to consumption sociology theory, consumption should be understood not only as an act of material exchange but also as a process of social identity formation and cultural expression. Consumption behaviors often carry symbolic meanings, through which individuals express identity, differentiate social status, and construct cultural taste within contemporary consumer culture [8]. Within the context of the She-Economy, women’s sports consumption vividly embodies this dual function of social distinction and self-expression. Contemporary women’s engagement in sports has transcended its original functional purpose of “physical exercise” and now represents a broader cultural pursuit of health, independence, and lifestyle aesthetics. Thus, sports consumption becomes an important means for women to construct self-identity and redefine their social roles within the evolving cultural and economic landscape.

Beyond explaining the cultural meaning of sports consumption, the theory also highlights its internal contradictions. However, consumption sociology theory also reveals the inherent contradictions behind this behavior. On the one hand, sports consumption is regarded as a symbol of women’s awakened self-awareness and growing subjectivity; on the other hand, it has been reshaped by the logic of capital into a form of “gendered consumption”. While women gain a sense of identity and agency through participation in sports consumption, they simultaneously become subject to renewed social expectations embedded in commercial narratives. Consequently, women’s sports consumption reflects a dual dilemma—being both empowered and disciplined within the intersecting frameworks of market ideology and gender norms.

### 3.2 Gender theory: Revealing the structural constraints of sports consumption

Gender theory originated from the feminist movements of the 1960s in Western societies and has since evolved into a comprehensive theoretical framework. It posits that gender is not merely a biological distinction but a socially constructed category. Through institutional arrangements, cultural narratives, and everyday practices, society continuously reinforces differences between men and women in terms of roles, identities, and behavioral norms, thereby establishing “male/female” as a structural social relationship. This socially constructed gender order permeates the field of sports and forms the deep structural background of women’s sports consumption. As Émile Durkheim stated in The Rules of Sociological Method, there exists a “social nature” in people’s ways of acting, thinking, and feeling—something external to individual consciousness—that exercises a form of coercive power over individuals regardless of their will. Under the influence of traditional gender ideologies, sport has historically been constructed as a male-dominated domain associated with masculinity, strength, and competition, while women have often been positioned as spectators or supporters rather than active participants [9]. Consequently, women’s participation and choices in sports consumption are constrained by this entrenched gendered cultural logic. Moreover, societal expectations surrounding the female body and aesthetics often lead women in fitness consumption to focus on external indicators such as “body shaping” or “fat reduction,” rather than on athletic performance or technical improvement [10]. These distinctions are not the result of individual preferences but rather reflect the operation of gender structures within the domain of sports consumption. Similar circumstances have been observed in other Global South contexts. Rind and Naz [11] point out that Pakistani women’s sports participation is shaped by the interplay between individual agency and socio-cultural constraints. This perspective provides useful insights for understanding women’s sports consumption beyond a market-oriented framework.

## 4. Motivational drivers in women’s sports consumption under the She-Economy

In examining the activation of women’s sports consumption motivations, the application of Colaizzi’s seven-step analysis method identified four main dimensions that stimulate women’s engagement in sports consumption: health motivation, social motivation, self-fulfillment motivation, and market-driven incentives (see Table 2).

**Table 2:** Progressive open coding and thematic abstraction of motivations stimulating women’s sports consumption.

Original Excerpts	Open Codes	Formulated Meaning	Theme
F3: *“Mainly because sports make me feel healthier”.* F1: *“I keep exercising mainly for health and to strengthen my body".*	improve health; maintain fitness; stress relief	Sports participation is motivated by the desire to improve physical and mental well-being.	B1 Health Motivation
*F2: “I feel that sports are part of my life. Many of my hobbies revolve around sports. When friends say, ‘Let’s try this activity’, I often impulsively buy a snowboard or hiking poles. I spend a lot on this kind of consumption”.* *F4: “Sometimes I join fitness or sports groups where I can meet many like-minded new friends”.*	peer influence; group participation; social interaction	Sports consumption is encouraged through social participation and interpersonal relationships.	B2 Social Motivation
F7: *“Besides the changes in my weight, sports make me feel a stronger sense of self and improve my quality of life”.*	self-worth; self-identity; lifestyle improvement	Sports participation contributes to identity construction and self-fulfillment.	B3 Self-Fulfillment Motivation
F6: *“I saw Yang Shuyu endorsing a sports brand. I thought the shoes she wore looked very comfortable, so I bought the same pair”.*	celebrity endorsement; online influence; brand attraction	Consumption decisions are influenced by digital marketing and celebrity endorsement.	B4 Market-Driven Incentives

### 4.1 Health motivation: Core needs for physical well-being and body shaping

Health serves as the most fundamental driving force behind women’s sports consumption. Several participants described health maintenance and physical well-being as important reasons for engaging in sports consumption. As one participant noted, *“I mainly keep exercising for my health and to strengthen my body”* (F1). Another participant explained that sports participation made her “*feel healthier*” (F3). This finding is also consistent with previous studies suggesting that regular physical activity contributes to both physical and psychological well-being [12], In a social context where “appearance is capital,” women’s pursuit of physical health through sport is almost inevitably tied to the accumulation of bodily capital—a body that appears disciplined, capable, and aligned with normative aesthetics. The process of shaping their bodies through exercise is thus not merely an act of self-expression but also a response to the social expectation that women must manage their appearance. They shape their bodies through exercise and experience joy during the process, especially by gaining recognition of their self-image and a sense of unique personal appeal. From the perspective of gender theory, this health-oriented motivation does not simply replace traditional ideals of “fragile beauty” with a pursuit of “healthy beauty.” Rather, under the influence of healthism, “health” itself has been aestheticized and moralized: a toned, fit body has become the new standard of feminine attractiveness, blurring the boundary between physical well-being and body-sculpting pressure.

At the same time, the psychological relaxation and stress relief brought by physical activity have become an important means for women to pursue mental well-being. As another participant shared, *“Every time I work out—whether it’s zoning out while lifting weights indoors or feeling that freedom when I’m standing on the surfboard—I feel totally relaxed”* (F2).“Exercise helps stabilize my mood a lot and also improves my sleep.”(F5).Notably, the sense of relaxation and emotional stability described by participants is inseparable from the anxiety that precedes it—anxiety about body shape, about failing to meet appearance standards, or about losing control over one’s life. Exercise becomes a ritual through which this anxiety is temporarily pacified, precisely because the act of working out reassures the individual that she is making progress toward the desired body and self. These findings suggest that women’s sports consumption motivations involve both physical health needs and psychological well-being.

### 4.2 Social motivation: Collective participation and expansion of social networks

Social motivation represents a key dimension in driving women’s sports consumption, as such consumption is not only a form of individual bodily practice but also an important platform for social interaction. Throughout their sports participation, women often build new social networks through fitness communities, sports clubs, and online workout check-in groups. Several participants described sports participation as an important way to connect with friends and expand social relationships. The social and emotional characteristics emphasized in the She-Economy are fully reflected in this process. For example, participant F2 explained that: *“For me, exercising is more like part of my daily life. Many of my hobbies revolve around sports. I’ll often end up buying a snowboard or a pair of trekking poles just because my friends say, ‘Let’s go try this activity,’ and I buy them on impulse. I make these kinds of purchases pretty often”* (F2). This narrative illustrates how sports consumption may be embedded within women’s everyday social interactions and collective participation experiences. Some participants also indicated that online fitness communities and social media platforms increased their opportunities for interaction and participation, while also enhancing emotional connection and social belonging [13]. On the other hand, sports consumption has become a means for women to expand their social networks and express their lifestyles [14]. Participant F4 similarly noted that joining fitness communities enabled her to *“meet many like-minded new friends”* (F4). Posting workout achievements on social media has increasingly become a strategy for women to establish and maintain social relationships [15]. Therefore, social motivation plays a significant and indispensable role in shaping women’s sports consumption behaviors.

### 4.3 Self-realization motivation: Awakening of agency and lifestyle choice

Several participants described sports participation as an important way to pursue self-growth, self-expression, and personal achievement [16]. For some participants, sports activities were not only forms of physical exercise, but also meaningful lifestyle choices associated with personal identity and emotional fulfillment [17]. Activities such as marathon running, rock climbing, and cycling were often described as ways to challenge personal limits and pursue self-development. As one participant described, *“I’ve been running marathons for a long time. Beyond the obvious physical changes, it’s really the sense of accomplishment I get from pushing myself over and over again that satisfies me mentally”* (F7). This narrative reflects how sports participation may provide women with a sense of achievement, self-confidence, and psychological fulfillment. This narrative illustrates how sports participation can provide women with a sense of achievement, self-confidence, and psychological fulfillment. Such self-realization-oriented motivation reflects a broader shift toward pursuing personal growth and self-development among women within the context of the She-Economy.

### 4.4 Market-driven incentives: Brand marketing, KOL influence, and digital fitness

Within the context of the She-Economy, commercial marketing and digital media have become important mechanisms through which women’s sports consumption is encouraged and shaped. Several participants indicated that brand marketing, celebrity endorsements, and online fitness content influenced their sports consumption decisions and exercise participation. As participant F8 noted, “Sometimes when I see a fitness vlogger I follow posting a new workout video, I can’t help wanting to move my body too” (F8). This narrative suggests that fitness influencers and social media content may stimulate women’s motivation for exercise and consumption through emotional resonance and everyday exposure. Meanwhile, this finding is also consistent with previous studies emphasizing the influence of KOLs and social media on women’s consumption intentions [18]. Fitness influencers and lifestyle creators disseminate workout routines and body-management concepts through short videos and social platforms, becoming important reference points for women’s consumption decisions. Furthermore, the widespread adoption of digital fitness apps, online workout programs, and sports-related e-commerce livestreaming [19] has significantly lowered participation barriers, enabling women to integrate physical activity more flexibly into their daily schedules. Participant F4 similarly described the convenience of digital fitness platforms: “I often follow my favorite online creators for workout routines—like Pamela’s videos. I can burn calories anytime, anywhere” (F4).

At the same time, digital fitness platforms do more than simply increase accessibility. By continuously presenting idealized female bodies, standardized workout routines, and algorithm-driven fitness content, they also reinforce socially constructed expectations regarding appearance, self-discipline, and body management. Thus, digital fitness simultaneously expands women’s opportunities for participation while reproducing subtle forms of gendered body surveillance. Participants were encouraged to exercise through online fitness communities, yet they were also repeatedly exposed to commercialized ideals of femininity that linked health with slimness, attractiveness, and continuous self-improvement. Consequently, digital fitness should be understood not merely as a technological facilitator, but as a socially mediated space where empowerment and discipline coexist.

## 5. Structural barriers and women’s negotiation of sports consumption under the She-Economy

The findings presented in this chapter suggest that women’s sports consumption under the She-Economy should not be understood simply as a process of increased consumption or expanding purchasing power. Rather, participants’ experiences reveal a dynamic interaction between individual aspirations and structural constraints. Although women actively pursued health, self-development, social recognition, and emotional well-being through sports consumption, these motivations were continuously negotiated within the constraints of family responsibilities, gender norms, market expectations, and institutional conditions(see Table 3). This perspective extends existing understandings of women’s sports consumption by highlighting the interaction between agency and structural conditions.

**Table 3:** Progressive open coding and thematic abstraction of structural barriers affecting women’s sports consumption.

Data Excerpt	Open Codes	Formulated Meaning	Theme
*F3: “For example, when my child gets out of school, I still need to help with homework and cook dinner, so it’s inconvenient to go to the gym. I prefer working out at home because I can use fragmented time”.* *F9:The commute takes too much time, so I have to rely on online fitness.*	lack of time; household duties; fragmented exercise time	Family and domestic responsibilities reduce opportunities for sports participation and create time-related constraints.	B5 Individual Level
*F3: “Gym membership fees are a bit high”.* *F9: “Badminton and the equipment—like rackets and accessories—are getting more and more expensive now”.*	high membership fees; expensive equipment; financial burden	Economic costs associated with sports participation limit women’s sports consumption behaviors.	
*F8: “Sometimes after work, when I go to the gym with friends or shop for sports gear instead of going home to cook or help my kid with homework, I always feel a bit guilty”.*	guilt from exercise; family expectation; domestic responsibility	Gender-role expectations within family relationships create emotional pressure and constrain sports participation.	B6 Interpersonal Level
*F9: “When we play badminton, the surrounding tables and courts—ping-pong or basketball—are mostly taken by men. There are very few women. Most areas are still male-dominated, so it feels a bit embarrassing to go there”.*	male-dominated space; embarrassment; lack of belonging	Gendered cultural norms and male-dominated sports environments create psychological discomfort for women.	
*F9: “Another reason I chose badminton is that in local gyms, most of the people there are men, and I don’t feel very comfortable in that environment”.*	uncomfortable gym environment; gender imbalance	Spatial structures within sports venues may discourage women’s participation.	B7 Structural Level
*F4: “I feel that there are still very few policies that specifically support women’s participation in sports or sports consumption”.*	lack of policy support; insufficient institutional attention	Limited policy and institutional support restrict women’s opportunities for sports participation and consumption.	

### 5.1 Individual level

#### 5.1.1 Temporal constraints and adaptive participation strategies in women’s sports participation.

The gendered distribution of unpaid care work remains the primary factor that consumes women’s leisure time. Social gender theory posits that women are socially assigned to the domain of childcare “because of their biological characteristics” and are often positioned outside public life or the “political sphere,” which reinforces the restrictive role of the homemaker. As a result, women frequently experience tensions when attempting to balance personal interests with family responsibilities. Despite changing gender norms, traditional expectations regarding women’s domestic responsibilities continue to influence their leisure time allocation. Women typically undertake the majority of housework, childrearing, and elder care, compared with men, women have far less discretionary time for self-improvement or leisure activities.

However, the findings indicate that time pressure does not necessarily lead to women’s complete withdrawal from sports consumption. Instead, participants demonstrated negotiated agency by actively adapting their exercise practices within existing time constraints. Rather than simply accepting the limitation imposed by competing work and family responsibilities, some women reorganized their daily routines, adopted fragmented exercise patterns, and utilized flexible digital fitness resources to maintain their engagement with sports activities. For example, participant F3 explained: ”For example, when my child gets out of school, I still need to help with homework and cook dinner, so it’s inconvenient to go to the gym. I just choose to work out at home because I can use fragmented time” (F3). This experience illustrates that women’s sports consumption is not merely determined by the availability of free time but involves continuous negotiation between structural expectations and individual aspirations. Home-based exercise and fragmented workouts represent adaptive strategies through which women transform limited temporal resources into opportunities for maintaining physical activity. In this sense, women’s participation reflects a process of negotiated agency, where they pursue health and self-care while simultaneously navigating the gendered distribution of domestic responsibilities.

#### 5.1.2 Economic constraints and selective consumption strategies in women’s sports participation.

Sports consumption often involves considerable financial investment, making economic cost an important structural constraint influencing women’s participation. Expenses such as annual gym memberships, yoga classes, personal training sessions, professional equipment, sportswear, and nutritional supplements may create economic thresholds that limit sustained engagement in certain forms of sports consumption. Several participants described sports-related expenses as a significant consideration when making participation decisions. As one participant noted, *“Private training fees are insanely expensive nowadays. Sometimes when I want to learn how to use a certain machine, I can only watch videos and figure it out myself”* (F4). This narrative reflects how high costs associated with professional instruction and fitness facilities may reduce women’s access to certain forms of sports participation. Participant F2 similarly explained, *“If I want to buy a board or take a class but my budget is tight, I have to choose one over the other”* (F2). This experience demonstrates that economic constraints shape not only whether women participate in sports consumption but also how they negotiate available choices. According to resource constraint perspectives, individuals or households with limited financial capacity tend to prioritize essential expenditures over discretionary forms of consumption, such as leisure and sports participation [20].

However, the findings suggest that financial limitations do not necessarily result in complete disengagement from sports. Instead, participants demonstrated negotiated agency by adopting selective consumption strategies [21], such as prioritizing functional products, delaying non-essential purchases, choosing more affordable alternatives, and utilizing free or low-cost exercise resources. Through these adaptive strategies, women actively balance personal aspirations for health, self-improvement, and sports participation with existing economic conditions.

### 5.2 Interpersonal level

#### 5.2.1 Gender-role expectations and women’s negotiated agency within social networks.

Although women’s economic independence has increased, traditional gender-role expectations remain highly resilient within families and intimate relationships. Several participants described how family expectations and interpersonal pressures influenced their sports participation and consumption behaviors. As one participant stated, *“Sometimes after work, when I go to the gym with friends or buy some sports gear instead of going home to cook, I feel really uneasy”* (F8). This narrative suggests that feelings of guilt were not merely individual emotional reactions, but reflected the internalization of traditional gender norms that associate women’s moral value with caregiving and domestic responsibility. Hochschild’s concept of the “second shift” highlights how women often continue to undertake disproportionate unpaid domestic labor after completing formal employment, resulting in unequal access to discretionary time for leisure and self-development. This unequal distribution of domestic labor not only limits women’s available time but also reinforces the expectation that women should prioritize family obligations over their own health and leisure. Existing research further suggests that women’s social class and life stage influence the amount of time and financial resources available for sports participation and related consumption [22]. Feminist sport scholars Costa and Guthrie further note that family roles constitute the primary barrier to women’s engagement in physical activity [23]. Nevertheless, these interpersonal pressures were not always expressed through direct restrictions; more often, they operated as subtle forms of emotional regulation that shaped women’s decisions and consumption practices.

Beyond family relationships, broader social expectations and peer attitudes also influenced women’s engagement with specific sports activities. As another participant recalled, “I was really into taekwondo fo*r a while, but I kept hearing comments like ‘girls aren’t suited for this’ or ‘it’s better not to learn taekwondo,’ and it confused and bothered me a lot”* (F4). This finding suggests that gender stereotypes regarding femininity and appropriate female behavior continue to influence women’s sports choices. In male-dominated cultural contexts, women in physically demanding or traditionally masculine sports may be perceived as deviating from feminine ideals of grace, quietness, and compliance, resulting in symbolic exclusion that discourages their entry into these spaces. These adaptive responses demonstrate that women’s sports participation was neither a simple act of resistance nor complete compliance with gender norms.

#### 5.2.2 Gendered sports spaces and women’s negotiated agency in participation.

From a broader socio-cultural perspective, women’s sports consumption behavior is also shaped by gender norms and gendered sports spaces. Several participants described feelings of discomfort and exclusion when entering male-dominated sports spaces. As one participant shared, *“When we play badminton, the surrounding ping-pong and basketball courts are mostly occupied by men. There are very few women. Most areas are still male-dominated, so it feels a bit embarrassing to go there”* (F9). This finding suggests that women may experience psychological discomfort in sports environments perceived as masculine or male-dominated. Participants’ discomfort was not simply an individual emotional response. Rather, it reflected the gendered spatial organization of many sports venues, where women often experienced heightened visibility as numerical minorities, reduced feelings of safety, and a limited sense of belonging. Under such conditions, women became more conscious of being observed, evaluated, or judged, particularly when participating in activities traditionally associated with men. Consequently, male-dominated sports environments may subtly discourage women’s participation by reinforcing cultural expectations regarding appropriate female behaviour and spatial boundaries. Extensive literature documents that since its earliest formation, sport has been marked by an “inherently male” hegemonic label [24]. Across different historical and cultural contexts, sporting practices have frequently reinforced male-centered norms, limiting women’s cultural legitimacy, visibility, and sense of belonging within sporting spaces [25].

Participants also described how body image concerns and appearance-related pressure influenced their willingness to participate in certain sports activities. As another participant noted, *“I always wanted to learn swimming, but I never tried because swimsuits show a lot of skin, and swimming pools are crowded. My body doesn’t look good, so I felt uncomfortable going”* (F8). This narrative illustrates how women’s sports participation may be influenced by body surveillance and appearance-related pressures. In highly visible sporting contexts, concerns about being observed, evaluated, or judged may reduce women’s confidence and willingness to participate. However, rather than completely withdrawing from these environments, participants actively adjusted their participation strategies. Some intentionally selected sports perceived as more gender-inclusive, exercised during less crowded periods, participated with friends, or relied on familiar communities to enhance their sense of comfort and belonging.

### 5.3 Structural level

#### 5.3.1 Industrial, market, and spatial constraints and adaptive strategies in women’s sports consumption.

At the structural level, women’s sports consumption within the wave of the She-Economy is constrained by a dual imbalance in both industry supply and market environments. On the industry side, a persistent “gender blind spot” and lack of genuine innovation remain evident. Several participants perceived that certain sports products targeted at women were priced higher despite limited functional differences. As one participant noted, “I’ve seen cases where the same sports shoes cost more just because the color or target group was different—it felt like a pink tax, and it bothered me” (F6). The so-called “pink tax”—originating from U.S. consumer tax practices in the 1930s—refers to the tendency for products targeted at women to be priced higher than comparable men’s products; once an item is given a female-preferred color or labeled as “for women,” its price often increases accordingly [26]. This finding suggests that some women perceive gender-targeted sports marketing as commercially exploitative rather than genuinely responsive to their needs. Participants also mentioned limitations in female-oriented sports services, including the lack of specialized training guidance and insufficient female coaching resources. Nevertheless, participants adapted their consumption choices according to the available facilities and services while actively adjusting their participation strategies to navigate existing market and spatial inequalities.

#### 5.3.2 Institutional constraints and women’s negotiated participation strategies in sports consumption.

Beyond industrial and spatial constraints, insufficient policy support and institutional provision—characterized by a persistent gender blindness in public service design. Several participants perceived that current sports-related policies and public services were tacitly built on a male-as-norm model, thus insufficiently addressing women’s specific needs in sports participation and consumption. Participants particularly emphasized the lack of flexible and gender-sensitive service arrangements within existing sports consumption systems. As one participant noted, *“I think companies, especially commercial gyms, could pay more attention to women. For example, when offering monthly memberships, they could consider women’s menstrual cycles and provide temporary suspension or extension options to protect women’s financial interests”* (F2). This finding reflects women’s expectations for more flexible and gender-responsive sports service systems that acknowledge physiological differences and life-cycle changes. Some participants also expressed the need for greater institutional support within workplaces and daily living environments. As another participant said, *“I think workplaces could provide fitness services for employees so that it’s more convenient for us to exercise”* (F3). This finding further indicates that institutional arrangements—presuming a male-as-norm, unencumbered participant—provide limited flexibility for women balancing employment, family care, and sports participation. Nevertheless, participants demonstrated negotiated agency by adapting their participation practices within existing institutional conditions.

### 5.4 Theoretical contributions to understanding women’s sports consumption under the She-Economy

This study extends existing research by demonstrating that women’s sports consumption under the She-Economy should be understood as a socially negotiated practice rather than simply a reflection of increased purchasing power or consumer autonomy. The findings reveal that women continuously negotiate between individual aspirations and structural constraints, suggesting that sports consumption constitutes a contradictory social field in which empowerment and constraint coexist.

From a gender-theoretical perspective, women’s sports consumption reflects a process of negotiated agency, through which participants actively respond to gender norms and structural barriers while pursuing health, identity construction, and emotional well-being. From the perspective of consumption sociology, sports consumption functions as a socially embedded practice that enables women to construct identities and seek social recognition while simultaneously responding to market expectations, commercial representations of femininity, and broader socio-cultural norms.

Based on these findings, a multilevel conceptual framework was developed to illustrate the dynamic interaction between motivational drivers, structural barriers, and negotiated agency in women’s sports consumption within the context of the She-Economy (Fig 1).

**Fig 1:**
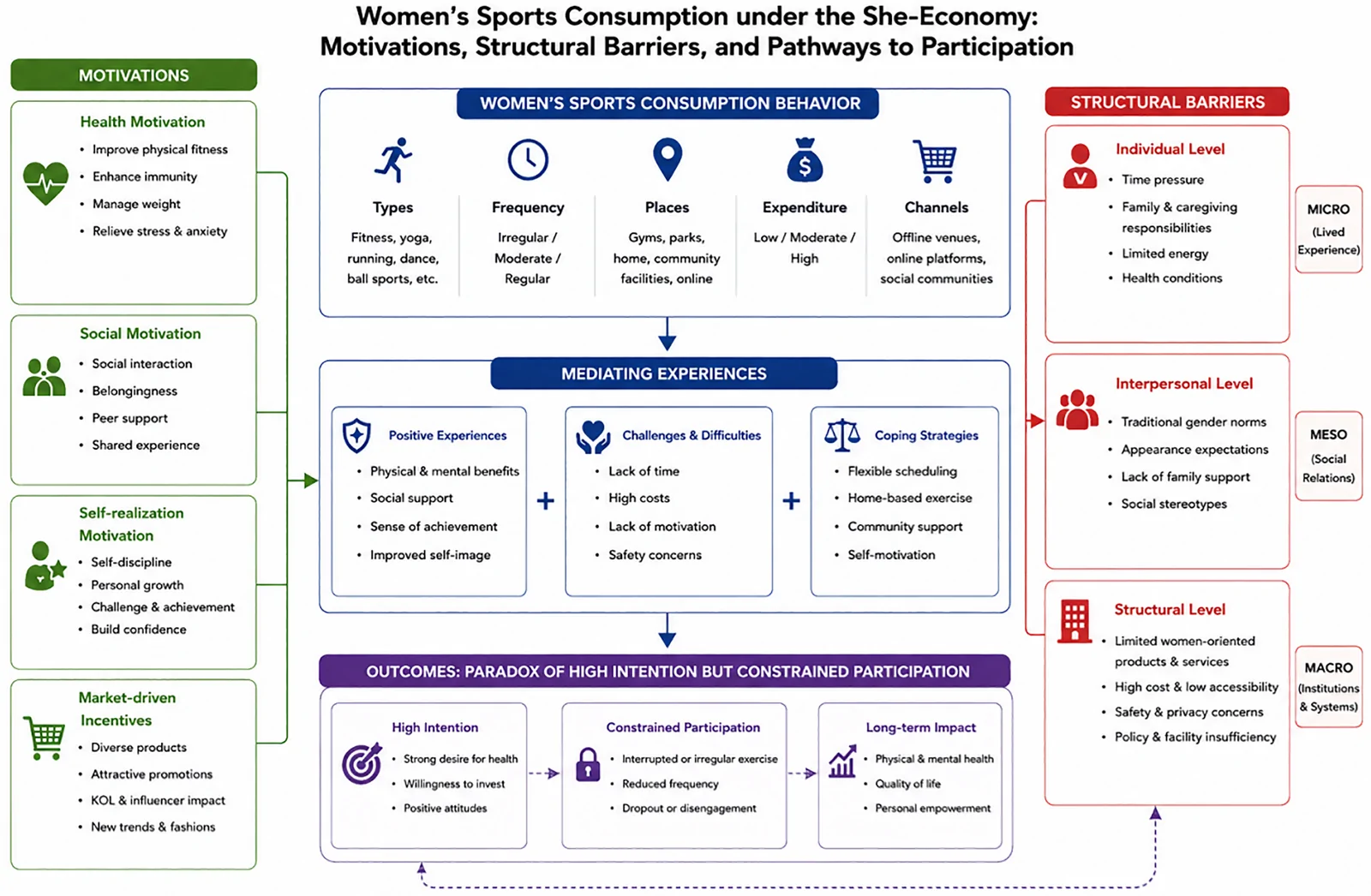
Multilevel framework of women’s sports consumption under the She-Economy.

## 6. Exploratory implications for women’s sports consumption based on the present findings

### 6.1 Exploratory insights for women-centered sports products and services

Participants in this study showed sensitivity to gendered pricing strategies and superficial product differentiation, while placing greater value on functionality, comfort, and practical usability in sports products. These reported preferences indicate that, within the experiences described by participants, sports products and services that emphasize functionality, comfort, and practical usability may better align with some women consumers’ expectations. Several participants expressed a preference for female trainers and personalized guidance, suggesting that such services may represent one possible approach to creating more supportive exercise environments in contexts similar to those described by participants.

For participants who experienced limited discretionary time due to work or family responsibilities, flexible and home-based sports consumption options appeared to provide practical alternatives. Against this backdrop, more flexible and home‑based sports consumption scenarios—such as digital fitness platforms, online workout programs, and home exercise options—may better accommodate women’s daily schedules by allowing physical activity to be integrated into limited time windows. Some participants also expressed preferences for more accessible and convenient exercise environments, suggesting that community-based or neighborhood-level sports spaces may represent one possible avenue for reducing participation difficulties in contexts similar to those described in this study. Flexible membership arrangements and family-supportive services may represent potential approaches for addressing the participation difficulties associated with family responsibilities described by some participants.

### 6.2 Exploratory implications for gender norms and social support in participants’ experiences

Participants’ experiences revealed that traditional gender expectations continued to shape how some women interpreted and negotiated their sports consumption practices. These experiences suggest that more gender-inclusive representations and supportive social narratives may be relevant for reducing some of the gendered pressures described by participants. For the participants in this study, more diverse and authentic portrayals of women’s sports participation may represent one possible way to challenge appearance-oriented understandings of women’s exercise.

At the family level, understanding and encouragement from spouses or other family members appear to influence women’s ability to maintain regular sports consumption [27]. Some participants indicated that when family members recognize the value of physical activity and actively share domestic duties, women are more likely to sustain their sports participation. Conversely, insufficient family support was described as increasing feelings of guilt and potentially weakening women’s persistence in sports participation. Among peers, mutual invitations, exercising together, and daily encouragement provide emotional motivation in an informal, non‑organized manner, lowering the psychological barrier for women to enter sports spaces alone. These informal support relationships appeared to function through everyday social interactions rather than relying exclusively on formal community structures or online platforms.

### 6.3 Implications for more gender-responsive sports services

Participants’ concerns extended beyond sports consumption itself to issues of accessibility, safety, and fairness within public sports services. The experiences reported by participants highlight the importance of considering gender-sensitive factors when designing sports services and facilities. Several participants expressed worries about safety, convenience, and privacy in existing public sports spaces. Therefore, future planning and renovation of public sports facilities might benefit from greater attention to the accessibility, privacy, and safety concerns described by participants, such as improving facility design and creating more comfortable exercise environments.

Participants’ experiences indicate that their sports consumption practices were shaped not only by individual motivations but also by institutional and market conditions. More responsive institutional support and service provision may help address some of the participation difficulties described by participants. Given participants’ concerns about the limited availability of women‑oriented sports products and services, future policy initiatives might consider providing financial or institutional incentives for enterprises developing women‑centered sports programs [28]. Meanwhile, flexible pricing strategies and more accessible service arrangements may represent potential approaches for addressing some economic constraints described by participants.

## 7. Conclusion

The findings demonstrate that women’s sports consumption is shaped through the continuous interaction between individual motivations and structural constraints, with participants adopting adaptive participation strategies to negotiate challenges related to time, family responsibilities, economic costs, gender norms, and institutional limitations. Rather than treating motivations and barriers as separate dimensions, this study conceptualizes women’s sports consumption as a socially negotiated practice in which empowerment and structural constraints coexist. These findings also provide exploratory implications for developing more inclusive sports environments and gender-sensitive support systems to support women’s sustained participation.

However, given the small qualitative sample and the context-specific nature of phenomenological research, the findings should be interpreted as exploratory rather than representative of all women consumers in China. Although gender was the primary analytical focus, participants’ narratives also suggest that women’s sports consumption is shaped by intersecting social positions. For example, participants with higher educational attainment and more flexible work schedules reported greater capacity to negotiate structural constraints, whereas those with greater caregiving responsibilities described more limited opportunities for sports participation. While the sample does not allow systematic subgroup comparisons, these findings highlight the potential influence of class, age, motherhood, and urban context. Future research should adopt an intersectional perspective to further examine how these dimensions jointly shape women’s sports consumption.

## Supporting information

S1 File Anonymized interview transcripts. Contains anonymized interview transcripts from all participants included in this study.
(ZIP)

S2 File Thematic analysis matrix (Colaizzi method). (DOCX)

S3 File Coding framework. (DOCX)

S4 File Interview guide. (DOCX)
