# Supplementary material for: Exploring the motivation and structural barriers of women’s sports consumption from the perspective of the “She-Economy”
Source: PLoS One. 2026 Oct 21;21(10):e0359201. doi: 10.1371/journal.pone.0359201 (PMC13686482; doi:10.1371/journal.pone.0359201)
Supplement: Supplementary file 1 [file pone.0359201.s002.docx]

Thematic Analysis Matrix (Colaizzi Method)

Purpose:

To transparently demonstrate how raw interview statements were transformed into formulated meanings, codes, and final themes.

| **Significant Statement** | **Formulated Meaning** | **Code** | **Theme** |
| --- | --- | --- | --- |
| *“I keep exercising mainly for health and to strengthen my body.” (F1)* | Sports consumption motivated by desire to improve physical well-being | H1 | Health Motivation |
| *“Exercise helps stabilize my mood a lot and also improves my sleep.” (F5)* | Exercise promotes mental well-being and emotional regulation | H2 | Health Motivation |
| *“For me, exercising is more like part of my daily life. Many of my hobbies revolve around sports. I’ll often end up buying a snowboard or a pair of trekking poles just because my friends say, ‘Let’s go try this activity,’ and I buy them on impulse. I make these kinds of purchases pretty often” (F2)* | Social influence stimulates consumption decisions | S1 | Social Motivation |
| *“Sometimes I join fitness or sports groups where I can meet many like-minded new friends.” (F4)* | Collective belonging reinforces participation and consumption | S2 | Social Motivation |
| *“I’ve been running marathons for a long time. Beyond the obvious physical changes, it’s really the sense of accomplishment I get from pushing myself over and over again that satisfies me mentally” (F7)* | Sports participation supports identity construction & self-worth | A1 | Self-Actualization |
| *“Sports are the way I express my attitude toward life.” (F6)* | Sports consumption represents self-expression and agency | A2 | Self-Actualization |
| *“I saw Yang Shuyu endorsing a sports brand. I thought the shoes she wore looked very comfortable, so I bought the same pair.” (F6)* | Brand endorsements influence purchasing behavior | M1 | Market Influence |
| *“Sometimes when I see a fitness vlogger I follow posting a new workout video, I can’t help wanting to move my body too.” (F8)* | Digital marketing stimulates consumption desire | M2 | Market Influence |
| *“For example, when my child gets out of school, I still need to help with homework and cook dinner, so it’s inconvenient to go to the gym. I prefer working out at home because I can use fragmented time.” (F3)* | Domestic responsibilities reduce ability to participate in sports | T1 | Time Constraints |
| *“The commute takes too much time, so I have to rely on online fitness.” (F9)* | Limited discretionary time restricts physical participation | T2 | Time Constraints |
| *“Badminton and the equipment—like rackets and accessories—are getting more and more expensive now.” (F9)* | High economic burden restricts consumption | E1 | Economic Barriers |
| *“Private training fees are insanely expensive nowadays. Sometimes when I want to learn how to use a certain machine, I can only watch videos and figure it out myself” (F4)* | Pricing of services limits accessibility | E2 | Economic Barriers |
| *“Sometimes after work, when I go to the gym with friends or buy some sports gear instead of going home to cook , I feel really uneasy” (F8)* | Gender norms pressure women to prioritize family over exercise | G1 | Gender Norms |
| *“Some people feel that strength training isn’t really suitable for girls.” (F5)* | Social expectations shape acceptable sports participation | G2 | Gender Norms |
| *“When we play badminton, the surrounding tables and courts—ping-pong or basketball—are mostly taken by men. There are very few women. Most areas are still male-dominated, so it feels a bit embarrassing to go there” (F9)* | Male-dominated fitness spaces create psychological exclusion | P1 | Spatial Barriers |
| *“Public sports facilities are not safe enough, so I avoid going at night.” (F5)* | Environmental insecurity discourages participation | P2 | Spatial Barriers |
| *“I feel that there are still very few policies that specifically support women’s participation in sports or sports consumption.” (F4)* | Policy gaps limit women’s access to sports participation | I1 | Institutional Barriers |
| *“There are no sports programs specifically designed to support women’s needs.” (F6)* | Lack of targeted institutional resource allocation | I2 | Institutional Barriers |
