# Supplementary material for: Exploring the motivation and structural barriers of women’s sports consumption from the perspective of the “She-Economy”
Source: PLoS One. 2026 Oct 21;21(10):e0359201. doi: 10.1371/journal.pone.0359201 (PMC13686482; doi:10.1371/journal.pone.0359201)
Supplement: Supplementary file 2 [file pone.0359201.s003.docx]

Coding Framework

Purpose:

This coding framework was developed following Colaizzi’s phenomenological analysis method to systematically interpret interview data and identify recurring patterns related to women’s sports consumption motivation and structural barriers.

| **Theme** | **Sub-theme** | **Code** | **Operational Definition** | **Example Quote (De-identified)** |
| --- | --- | --- | --- | --- |
| **Motivations for Women’s Sports Consumption** | Health motivation | H1 | Sports participation driven by desire to improve physical health, prevent illness, or maintain strength | “I mainly exercise to stay healthy and strengthen my body.” (F1) |
|  | Body shaping & appearance | H2 | Exercise aimed at weight control, fitness aesthetics, or enhancing physical attractiveness | “Working out makes me feel better about my figure.” (F5) |
|  | Social interaction | S1 | Consumption influenced by friends, peer groups, or social belonging | “When friends invite me to try a sport, I usually buy the equipment and go with them.” (F2) |
|  | Community identity | S2 | Joining or forming exercise groups to build social networks and shared identity | “I meet many like-minded women in fitness communities.” (F4) |
|  | Self-actualization | A1 | Sports participation as personal empowerment, autonomy, confidence, or psychological fulfillment | “Running marathons helps me find my sense of agency.” (F7) |
|  | Lifestyle expression | A2 | Exercise used to communicate one’s values, personality, or modern lifestyle | “Sports are part of who I am now.” (F8) |
|  | Market influence | M1 | Purchases triggered by brand marketing, celebrity endorsement, KOL recommendations | “I bought a pair because my favorite athlete endorsed that brand.” (F6) |
|  | Digital fitness incentives | M2 | Sports consumption driven by online platforms, apps, or livestream fitness | “I follow online workout videos at home and end up buying related products.” (F4) |
| **Structural Barriers to Women’s Sports Consumption** | Time constraints | T1 | Lack of disposable time due to work, caregiving, housework, or multitasking | “After helping my child with homework and cooking, there’s no time to go to the gym.” (F3) |
|  | Economic burden | E1 | Sports consumption limited by high costs of classes, equipment, facilities | “Gym memberships and private lessons are too expensive.” (F9) |
|  | Family role expectations | G1 | Internalized pressure to prioritize family obligations over personal exercise | “If I don’t go home to cook, I feel guilty for spending time at the gym.” (F8) |
|  | Social gender norms | G2 | Cultural beliefs discouraging women from participating in certain sports | “People said girls shouldn’t learn taekwondo, which confused me.” (F4) |
|  | Male-dominated sports spaces | P1 | Discomfort or withdrawal due to male gaze, spatial exclusion, gym culture | “Most people at the gym are men, so I feel uneasy working out there.” (F9) |
|  | Body surveillance anxiety | P2 | Concern about being judged for physical appearance in public exercise spaces | “I avoided swimming because I worried about being seen in swimwear.” (F8) |
|  | Inadequate market supply | S3 | Products/services failing to address women's physiological and practical needs | “Sometimes there’s no product that really fits what women need.” (F2) |
|  | Institutional & policy gaps | I1 | Insufficient public support, workplace programs, or gender-informed sports policies | “There aren’t many policies encouraging women to exercise.” (F4) |
