# Supplementary material for: Exploring the motivation and structural barriers of women’s sports consumption from the perspective of the “She-Economy”
Source: PLoS One. 2026 Oct 21;21(10):e0359201. doi: 10.1371/journal.pone.0359201 (PMC13686482; doi:10.1371/journal.pone.0359201)
Supplement: Supplementary file 3 [file pone.0359201.s004.docx]

Interview Guide

Study Title：Exploring the Motivation and Structural Barriers of Women’s Sports Consumption under the “She-Economy”

Purpose of the Interview：

This interview aims to explore women’s motivations, decision-making processes, consumption experiences, and perceived structural barriers related to sports participation and sports consumption.

Interview Format

1.Semi-structured, one-on-one interview

2.Approximate duration: 20–40 minutes

3.Conducted in person or online audio/video call

4.Audio-recorded with participant’s informed consent

Opening Statement & Informed Consent

“Hello, thank you for participating in this research interview. I am a graduate student conducting a study on women’s sports consumption. The interview aims to understand your real experiences, thoughts, and feelings related to sports participation and consumption. Your responses will remain confidential and used solely for academic purposes. All personal identifiers will be removed during transcription. Participation is voluntary, and you may skip any question or stop at any time. With your permission, I would like to audio-record the interview to ensure accuracy. Do you agree to participate and to be recorded?”

Section 1 — Warm-Up & Behavioral Background

1.When did you begin regularly participating in sports or fitness activities?

2.What does your current exercise routine look like? (frequency, type, location)

3.What kinds of sports-related spending do you typically make?

Probe: “Can you recall a recent purchase that you felt was worthwhile?”

Section 2 — Motivations & Consumption Decisions

1.What underlying reasons motivate you to continue investing time and money in sports participation?

Probe: health, appearance, emotional release, habit, discipline

2.What role does sports participation play in your social life?

Probe: friends, communities, exercise partners, online groups, influencers

3.Besides physical changes, what psychological or emotional benefits do you gain from exercising?

4.When choosing sports products or services, what factors influence your decisions?

Probe: price, quality, function, brand image, instruction quality, environment

Section 3 — Perceived Barriers & Challenges

1.Have you ever experienced time pressure or financial constraints when engaging in sports consumption?

Probe: childcare, work schedule, training costs, gym fees

2.How do your family members, partner, or close social circle view your sports consumption?

Probe: support, discouragement, expectations

3.Have you ever felt uncomfortable exercising in public settings (e.g., gyms, parks)?

Probe: gendered spaces, being watched, body image concerns

4.Do you feel the current sports market adequately meets women’s needs?

Probe: product design, facility layout, coaching, safety, inclusiveness

Section 4 — Reflections & Recommendations

1.In your opinion, what could society, families, or companies do to better support women’s sports participation?

2.Is there anything else you feel is important that we have not discussed?

Closing Statement

“Thank you again for your time and valuable insights. Your contribution is extremely meaningful to this research. Wish you all the best.”
