# Supplementary material for: Exploring the motivation and structural barriers of women’s sports consumption from the perspective of the “She-Economy”
Source: PLoS One. 2026 Oct 21;21(10):e0359201. doi: 10.1371/journal.pone.0359201 (PMC13686482; doi:10.1371/journal.pone.0359201)
Supplement: Supplementary file 4 [file pone.0359201.s001.zip › Interview Transcript 1.docx]

Interview Transcript 1

Appendix I: Participant Information Sheet

(Used for participant recruitment and screening prior to the interview, and for describing the sample composition in the study)

Interview ID: F1 (To protect privacy, participant names are replaced with codes such as F01, F02, etc.)

Interview Method:

☑ Online Telephone Interview

□ Face-to-Face Interview

I. Demographic Information

Age: 50

Occupation: Homemaker

Education Level:

□ High School or Below

☑ Associate Degree

□ Bachelor's Degree

□ Master's Degree or Above

Average Monthly Income (Optional):

□ Below RMB 3,000

□ RMB 3,001–6,000

□ RMB 6,001–10,000

□ RMB 10,001–15,000

□ RMB 15,001–20,000

□ Above RMB 20,000

II. Sports Consumption Characteristics

6. What are the one or two sports you participate in most frequently?

Badminton

7. Approximately how much do you spend on sports-related activities each month? (Including equipment, courses, memberships, sports events, etc.)

□ Below RMB 200

☑ RMB 201–500

□ RMB 501–1,000

□ RMB 1,001–2,000

□ Above RMB 2,000

8. What are your main types of sports consumption? (Multiple choices allowed)

☑ Sports Equipment (clothing, shoes, gear)

□ Gym/Studio Membership

□ Personal Training

□ Group Classes (Yoga, Pilates, Aerobics, etc.)

□ Sports Event Tickets

□ Online Fitness App Membership

□ Other: __________

Appendix II: Interview Content

Interviewer (I): Hello, thank you for participating in this academic interview. I am currently pursuing postgraduate studies at a university in Northwest China. I am conducting a research project on women’s sports consumption.

The purpose of this interview is to understand your experiences, perceptions, and thoughts regarding sports consumption as a woman.

All information will remain confidential and be used solely for academic purposes. Any information included in future publications will be anonymized.

The interview is expected to last approximately five to six minutes. If at any point you feel uncomfortable with a question, you may skip it or terminate the interview. To ensure accuracy, I would like to record our conversation. Do I have your permission?

Respondent (R): Yes, that's fine.

I: Let's begin. When did you start participating regularly in sports or fitness activities?

R: Probably around 2021. In fact, I have maintained an exercise habit for quite a long time.

I: What does your current exercise routine look like? For example, the frequency, type of sport, and where you usually exercise.

R: Mostly at the local sports center.

I: What kind of sports do you mainly participate in?

R: Badminton. I practice badminton every morning.

I: What are your main sports-related expenditures?

R: Mostly badminton equipment and related gear.

I: Have you ever enrolled in sports classes or joined a gym?

R: No.

I: What was the most recent sports-related purchase that you felt was particularly worthwhile?

R: Still badminton-related purchases, mainly equipment.

I: What motivates you to continue investing your time and money in sports? Is it for health, appearance, stress relief, or something else?

R: I keep exercising mainly for health and to strengthen my body.

I: What role does sports play in your social life?

Have you ever made sports-related purchases because of friends, influencers, celebrities, or online recommendations?

R: Not really. I mostly make decisions myself based on my own habits. I am quite self-disciplined and prefer offline consumption.

I: Besides physical changes, what psychological or emotional benefits has exercise brought to you?

R: It makes me feel refreshed and energetic throughout the day.

I: What factors do you value most when purchasing sports products or services? Is it the brand, design, functionality, or something else? Could you give an example?

R: Mainly brand preference. Once I get used to a particular brand, I tend to stick with it and rarely switch.

I: Have you encountered any time-related or financial difficulties while maintaining your sports consumption? How have you dealt with them?

R: No. Both my time and financial situation are relatively flexible, so sports-related spending does not create much pressure for me.

I: What is your family's or spouse's attitude toward your sports participation and sports consumption?

R: They are all very supportive.

I: Have family responsibilities, such as caring for children, ever affected your exercise plans?

R: No.

I: Have you ever felt uncomfortable or embarrassed in gyms or other public sports spaces?

R: No. I generally feel quite comfortable in those environments.

I: Have you ever used specific exercise areas, such as free-weight zones? Do you think these areas are designed more for men than for women?

R: Not particularly. I think women are also taken into consideration, and overall the environment is quite acceptable.

I: Do you worry about others judging your body shape or exercise movements while you are exercising?

R: Not really. Perhaps because my movements are generally quite standard.

I: Do you think current sports products and services adequately meet women's needs?

R: Yes, I am very satisfied.

I: Do you feel there are any products or services missing from the market that you would like to have?

R: No. The current market basically meets my everyday exercise needs.

I: In your opinion, what could society, families, or related enterprises do to better support women in enjoying sports?

R: They could organize more activities to help women maintain exercise habits, encourage more women to participate, and allow them to experience the enjoyment of sports.

I: Is there anything else you would like to share, or any important topic related to women's sports consumption that I have not asked about?

R: No, I think that's all.

I: Thank you. Those are all my questions. Once again, thank you for taking the time to share your experiences and thoughts. I wish you all the best.

R: Thank you.
