# Supplementary material for: Exploring the motivation and structural barriers of women’s sports consumption from the perspective of the “She-Economy”
Source: PLoS One. 2026 Oct 21;21(10):e0359201. doi: 10.1371/journal.pone.0359201 (PMC13686482; doi:10.1371/journal.pone.0359201)
Supplement: Supplementary file 4 [file pone.0359201.s001.zip › Interview Transcript 2.docx]

Interview Transcript 2

Appendix I: Participant Information Sheet

(Used for participant recruitment and screening prior to the interview, and for describing the sample composition in the study)

Interview ID: F2 (To protect privacy, participant names are replaced with codes such as F01, F02, etc.)

Interview Method:

☑ Online Telephone Interview

□ Face-to-Face Interview

I. Demographic Information

Age: 27

Occupation: Self-Media Content Creator

Education Level:

□ High School or Below

□ Associate Degree

□ Bachelor's Degree

☑ Master's Degree or Above

Average Monthly Income (Optional):

□ Below RMB 3,000

□ RMB 3,001–6,000

□ RMB 6,001–10,000

□ RMB 10,001–15,000

□ RMB 15,001–20,000

□ Above RMB 20,000

II. Sports Consumption Characteristics

6. What are the one or two sports you participate in most frequently?

Surfing, Skiing

7. Approximately how much do you spend on sports-related activities each month? (Including equipment, courses, memberships, sports events, etc.)

□ Below RMB 200

☑ RMB 201–500

□ RMB 501–1,000

□ RMB 1,001–2,000

□ Above RMB 2,000

8. What are your main types of sports consumption? (Multiple choices allowed)

☑ Sports Equipment (clothing, shoes, gear)

☑ Gym/Studio Membership

☑ Personal Training

□ Group Classes (Yoga, Pilates, Aerobics, etc.)

□ Sports Event Tickets

□ Online Fitness App Membership

□ Other: __________

Appendix II: Interview Content

Interviewer (I): Hello, thank you for participating in this academic interview. My name is Shiyi Xuan, and I am a postgraduate student in Sports Humanities and Sociology. I am conducting a research project on women’s sports consumption. The purpose of this interview is to understand your experiences, perceptions, and feelings regarding sports consumption as a woman. All information will remain confidential and will be used solely for academic purposes. Any information included in future publications will be anonymized. The interview will last approximately ten minutes. If you feel uncomfortable with any question, you may skip it or stop the interview at any time. To ensure accuracy, I would like to record our conversation. Do I have your permission?

Respondent (R): Yes, I agree.

I: Great. Could you tell me when you first began participating regularly in sports or fitness activities?

R: I started about three and a half to four years ago. At the beginning, I was mainly involved in traditional fitness and bodybuilding-related activities.

I: That is quite a long period of consistent participation. What does your current exercise routine look like? Could you describe the frequency, type of sports, and locations where you usually exercise?

R: There are generally two situations. For indoor activities, I usually do strength training in a conventional gym. During the summer, I train four to five times a week. In winter, I am a bit more conservative and usually train around three times a week.

For outdoor activities, I prefer extreme sports such as skiing and surfing. These sports are highly seasonal, so I usually participate during the surfing season, around May and June, and the skiing season, around December and January. Each season lasts about two months.

I: What are your primary sports-related expenditures?

R: First of all, outdoor sports equipment. Recently, I have spent a lot on skis and skiing gear. These purchases provide a very tangible sense of experience and enjoyment. I also spend quite a lot on training courses, such as hiring coaches and purchasing lessons.

I: Do you invest money in sportswear as well?

R: Absolutely. I believe that good sportswear significantly improves my exercise experience. Therefore, I spend a considerable amount on skiing equipment, surfboards, fitness clothing, and even small accessories.

I: So you feel that these expenses are worthwhile?

R: Definitely.

I: What motivates you to continue investing time and money in sports? Is it for health, appearance, stress relief, or something else?

R: I would say stress relief is the biggest reason. Modern life, study, and work all come with considerable pressure.

R: “Every time I work out—whether it’s zoning out while lifting weights indoors or feeling that freedom when I’m standing on the surfboard—I feel totally relaxed.”

R: That feeling of relaxation is probably my strongest internal motivation for continuing to exercise.

I: So stress relief is the primary reason?

R: Yes, exactly.

I: What role does sports play in your social life? Have you ever made sports-related purchases because of friends, communities, or influencers?

R: Definitely. Sports have become an important part of my lifestyle.

R: “For me, exercising is more like part of my daily life. Many of my hobbies revolve around sports. I’ll often end up buying a snowboard or a pair of trekking poles just because my friends say, ‘Let’s go try this activity,’ and I buy them on impulse. I make these kinds of purchases pretty often.”

I: Besides physical changes, what emotional or psychological benefits has sports participation brought you?

R: Sports allow me to meet more like-minded people. They have made me more outgoing and sociable, while also helping me relieve stress effectively.

I: When purchasing sports products or services, what factors matter most to you? Brand, design, functionality, coaches, or atmosphere? Could you give an example?

R: Taking fitness classes as an example, I first evaluate the gym facilities. I want to know whether the equipment is comprehensive and whether it can support the specific training goals I have. Secondly, I pay close attention to the coach’s professionalism. If a coach is highly professional and makes the sessions enjoyable, I will continue purchasing classes from them. However, if I feel the coach lacks expertise or the sessions become boring, I would consider changing either the coach or the gym. Overall, service quality is what I value most.

I: Have you encountered any time-related or financial difficulties while maintaining sports consumption? How do you manage them?

R: Quite often.

R: “If I want to buy a board or take a class but my budget is tight, I have to choose one over the other.”

R: In most cases, I still prioritize spending on sports-related activities.

Regarding time, if I have a meeting in the afternoon but also want to work out, I will try to compress my work schedule to ensure that I still have time for exercise.

I: So even when facing time or financial constraints, sports tend to remain a priority?

R: Yes, exactly.

I: What is the attitude of your family or partner toward your sports participation and sports-related spending?

R: My parents, family members, and partner are all very supportive. I do not have children at the moment, but if I do in the future, I would like to exercise together with them and encourage them to participate in sports as well.

I: Have you ever felt uncomfortable or embarrassed in public sports spaces such as gyms or parks? What happened?

R: Yes. When I first started going to the gym, I was unfamiliar with some of the equipment and accidentally got hit by a machine once. That was quite embarrassing.

I: Do you think certain sports spaces, such as free-weight areas, are designed more for men? How do you feel about that?

R: To some extent, yes. Many gyms still seem to cater primarily to men. Similarly, when I take skiing or surfing lessons, the coaches are almost always male. Because these sports inevitably involve some physical contact during instruction, that can feel somewhat unfriendly to women who are uncomfortable with such interactions.

I: Do you worry about others judging your body shape or exercise performance?

R: Not really. I actually think it is beneficial when others point out areas where I can improve. I enjoy communicating with people at the gym and exchanging advice.

I: Do you think current sports products and services adequately meet women's needs? Are there any products or services you feel are missing?

R: Honestly, there is still room for improvement. For example, in the area of protein supplements, it would be great to have products specifically designed for women that take hormonal characteristics into account. With skis, in addition to height and weight considerations, it would be nice if manufacturers also paid more attention to women's aesthetic preferences. Furthermore, items such as dumbbells and gym equipment could be designed with greater consideration for women's hand sizes, fingernails, and other practical details.

I: Looking back on your experiences, what do you think society, families, or businesses could do to better support women in enjoying sports?

R: “I think companies, especially commercial gyms, could pay more attention to women. For example, when offering monthly memberships, they could consider women’s menstrual cycles and provide temporary suspension or extension options to protect women’s financial interests.”

I: Is there anything else you would like to share, or any topic that I have not asked about but that you believe is important for understanding women’s sports consumption?

R: I would like to say that contemporary women should not be confined to traditional spaces such as the home or the kitchen. Women should be free to appear wherever they choose—whether in sports, entertainment, or any other sphere of life. I hope society will pay greater attention to women, respect women, and contribute to their freedom, empowerment, and continued advancement.

I: Thank you very much. Those are all my questions. Once again, I sincerely appreciate your time and willingness to share your experiences and perspectives. Your insights are extremely valuable for my research. I wish you all the best.

R: Thank you.
