# Supplementary material for: Exploring the motivation and structural barriers of women’s sports consumption from the perspective of the “She-Economy”
Source: PLoS One. 2026 Oct 21;21(10):e0359201. doi: 10.1371/journal.pone.0359201 (PMC13686482; doi:10.1371/journal.pone.0359201)
Supplement: Supplementary file 4 [file pone.0359201.s001.zip › Interview Transcript 3.docx]

Interview Transcript 3

Appendix I: Participant Information Sheet

(Used for participant recruitment and screening prior to the interview, and for describing the sample composition in the study)

Interview ID: F3 (To protect privacy, participant names are replaced with codes such as F01, F02, etc.)

Interview Method:

□ Online Video Conference

☑ Face-to-Face Interview

I. Demographic Information

Age: 47

Occupation: Public Institution Employee

Education Level:

□ High School or Below

□ Associate Degree

☑ Bachelor's Degree

□ Master's Degree or Above

Average Monthly Income (Optional):

□ Below RMB 3,000

□ RMB 3,001–6,000

☑ RMB 6,001–10,000

□ RMB 10,001–15,000

□ RMB 15,001–20,000

□ Above RMB 20,000

II. Sports Consumption Characteristics

6. What are the one or two sports you participate in most frequently?

Jump Rope, Online Group Fitness Workouts

7. Approximately how much do you spend on sports-related activities each month? (Including equipment, courses, memberships, sports events, etc.)

☑ Below RMB 200

□ RMB 201–500

□ RMB 501–1,000

□ RMB 1,001–2,000

□ Above RMB 2,000

8. What are your main types of sports consumption? (Multiple choices allowed)

☑ Sports Equipment (clothing, shoes, gear)

□ Gym/Studio Membership

□ Personal Training

☑ Group Classes (Yoga, Pilates, Aerobics, etc.)

□ Sports Event Tickets

□ Online Fitness App Membership

□ Other: __________

Appendix II: Interview Content

Interviewer (I): Hello, thank you for participating in this academic interview. My name is Shiyi Xuan, and I am a postgraduate student in Sports Humanities and Sociology. I am conducting a research project on women’s sports consumption. The purpose of this interview is to understand your real experiences, thoughts, and feelings regarding sports consumption as a woman. All information will remain confidential and will be used solely for academic purposes. Any information included in future publications will be anonymized. The interview will last approximately ten minutes. If you feel uncomfortable with any question, you may skip it or terminate the interview at any time. To ensure accuracy, I would like to record our conversation. Do I have your permission?

Respondent (R): Yes, I agree.

I: Great. Let’s begin. When did you start participating regularly in sports or fitness activities?

R: I started exercising regularly around 2018 and have maintained the habit ever since.

I: What does your current exercise routine look like? For example, the frequency, activities, and locations.

R: I mainly exercise at home. My activities are relatively simple, such as hula hoop workouts and jump rope exercises.

I: What are your main sports-related expenditures?

R: Mostly sportswear and fitness clothing.

I: Any sports equipment as well?

R: Yes, things like hula hoops and jump ropes. They are relatively simple and inexpensive.

I: After maintaining these exercise habits, have you noticed any changes? What motivates you to continue investing time and money in sports?

R: The main reason is health. Exercise helps me stay physically healthy and improves my body shape. It also helps relieve work-related stress.

I: What role does sports play in your social life? Do your friends or colleagues also exercise regularly?

R: Yes. Many of my colleagues and friends exercise regularly. Some go swimming, some attend group fitness classes, and others practice yoga.

I: When purchasing sports products or services, what factors matter most to you? Brand, design, functionality, or atmosphere?

R: I pay more attention to functionality. I always look for products that are practical and cost-effective.

I: Have you encountered any time-related or financial difficulties while maintaining sports consumption?

R: Yes.

R: “Gym membership fees are a bit high.”

R: In addition, gyms are not always convenient for me because of family responsibilities.

I: So family and financial factors influence your choices?

R: Yes.

I: Have family responsibilities, such as taking care of children, ever affected your exercise plans?

R: Yes, definitely.

R: “For example, when my child gets out of school, I still need to help with homework and cook dinner, so it's inconvenient to go to the gym. I just choose to work out at home because I can use fragmented time.”

R: Home-based exercise allows me to fit physical activity into my daily routine much more easily.

I: Have you ever felt uncomfortable or embarrassed in public sports spaces such as gyms or parks?

R: Yes. There tend to be more men in gyms, and sometimes there are very few women around, so I do not participate in those environments very often.

I: Do you worry about other people judging your body shape or exercise performance?

R: No, not really.

I: Do you think current sports products and services adequately meet women’s needs? Are there any products or services you feel are lacking?

R: Overall, I am quite satisfied. Most products and services can meet my exercise needs.

I: Looking back on your experiences, what do you think society, families, or related organizations could do to better support women’s participation in sports?

R: “I think workplaces could provide fitness services for employees so that it’s more convenient for us to exercise.”

R: If exercise opportunities were available through workplaces, it would be much easier for many women to maintain regular physical activity.

I: Is there anything else you would like to share?

R: No, I think that covers everything.

I: Thank you very much for sharing your experiences and perspectives. Your insights are extremely valuable for this research. I wish you all the best.

R: Thank you.
