# Supplementary material for: Exploring the motivation and structural barriers of women’s sports consumption from the perspective of the “She-Economy”
Source: PLoS One. 2026 Oct 21;21(10):e0359201. doi: 10.1371/journal.pone.0359201 (PMC13686482; doi:10.1371/journal.pone.0359201)
Supplement: Supplementary file 4 [file pone.0359201.s001.zip › Interview Transcript 4.docx]

Interview Transcript 4

Appendix I: Participant Information Sheet

(Used for participant recruitment and screening prior to the interview, and for describing the sample composition in the study)

Interview ID: F4 (To protect privacy, participant names are replaced with codes such as F01, F02, etc.)

Interview Method:

□ Online Video Conference

☑ Face-to-Face Interview

I. Demographic Information

Age: 24

Occupation: Student

Education Level:

□ High School or Below

□ Associate Degree

□ Bachelor's Degree

☑ Master's Degree or Above

Average Monthly Income (Optional):

□ Below RMB 3,000

☑ RMB 3,001–6,000

□ RMB 6,001–10,000

□ RMB 10,001–15,000

□ RMB 15,001–20,000

□ Above RMB 20,000

II. Sports Consumption Characteristics

6. What are the one or two sports you participate in most frequently?

Swimming, Badminton

7. Approximately how much do you spend on sports-related activities each month? (Including equipment, courses, memberships, sports events, etc.)

□ Below RMB 200

□ RMB 201–500

☑ RMB 501–1,000

□ RMB 1,001–2,000

□ Above RMB 2,000

8. What are your main types of sports consumption? (Multiple choices allowed)

☑ Sports Equipment (clothing, shoes, gear)

☑ Gym/Studio Membership

☑ Personal Training

□ Group Classes (Yoga, Pilates, Aerobics, etc.)

□ Sports Event Tickets

□ Online Fitness App Membership

□ Other: __________

Appendix II: Interview Content

Interviewer (I): Hello, thank you for participating in this interview. My name is Shiyi Xuan, and I am currently conducting research on women’s sports consumption. The interview content will be used solely for academic purposes, and all information will be anonymized. Do I have your permission to record this interview?

Respondent (R): Yes, I agree.

I: When did you start participating in sports regularly?

R: Probably during my university years. At first, I wanted to lose weight because I felt I had gained some weight. Later, during the pandemic, I was exposed to some online fitness programs and gradually developed a regular exercise habit.

I: What are your current exercise habits?

R: I mainly swim and do home-based fitness workouts. When the weather is good, I go swimming two or three times a week. At home or in my dormitory, if space allows, I often follow Pamela’s workout videos.

I: Why did you choose Pamela’s workouts?

R: They are convenient and quite effective. I follow several international fitness influencers, and Pamela is definitely my favorite. I often exercise following her videos.

R: “I often follow my favorite online creators for workout routines—like Pamela’s videos. I can burn calories anytime, anywhere.”

R: For students like me, it is difficult to maintain a fixed gym schedule, so online workouts are extremely convenient. I can exercise whenever and wherever I want.

I: What are your major sports-related expenditures?

R: Mainly swimsuits, goggles, sportswear, and swimming pool admission fees. Occasionally, I also purchase online courses or fitness app memberships.

I: What was the most worthwhile sports-related purchase you made recently?

R: A professional swimsuit. I used to buy cheaper ones, but they were uncomfortable. After switching to a higher-quality brand, my swimming experience improved significantly.

I: What motivates you to continue investing time and money in sports?

R: Health is definitely one reason, but more importantly, exercise helps me improve my overall well-being. I feel happier, more energetic, and more confident after working out.

I: What role does sports play in your social life?

R: It plays a fairly important role. I’ve met many people who share similar interests in sports.

R: Sometimes I join fitness or sports groups where I can meet many like-minded new friends.

I: Do these communities influence your consumption decisions?

R: Yes, they do. People often recommend sports equipment, share experiences with different courses, and suggest good sports venues. Before purchasing something, I often ask for advice from friends in these groups.

I: Besides physical changes, what psychological or emotional benefits have you gained from exercise?

R: The biggest change is that I’ve become much more confident. In the past, I paid a lot of attention to my appearance and body shape. Now, I focus more on whether my physical abilities are improving.

I: What factors do you value most when purchasing sports products or services?

R: Cost-effectiveness and functionality. As a student, my budget is limited, so I pay close attention to value for money.

I: Have you ever considered purchasing personal training services?

R: Yes, I have, but they are too expensive.

R: “Private training fees are insanely expensive nowadays. Sometimes when I want to learn how to use a certain machine, I can only watch videos and figure it out myself.”

R: Most of the time, I have to learn by watching videos online or searching for tutorials myself.

I: What difficulties have you encountered while maintaining your sports participation?

R: Time and financial constraints are both factors. As a student, my income is limited, so I have to think carefully before making sports-related purchases.

I: What is your family’s attitude toward your sports participation and sports consumption?

R: They are generally supportive. They believe exercise is beneficial for my health.

I: Have you experienced any interpersonal barriers related to sports participation?

R: Yes.

I: Could you elaborate?

R: I was very interested in taekwondo for a period of time and even attended some trial classes.

R: However, people around me often made comments such as “girls are not suited for this” or “it’s better not to learn taekwondo.”

R: “I was really into taekwondo for a while, but I kept hearing comments like ‘girls aren’t suited for this’ or ‘it’s better not to learn taekwondo,’ and it confused and bothered me a lot.”

R: Although those comments were not necessarily intended to be harmful, they made me question whether I was truly suitable for the sport.

I: Did those experiences influence your sports choices?

R: To some extent, yes. Eventually, I stopped pursuing taekwondo in a systematic way.

I: Do you worry about being judged based on your body shape or athletic performance?

R: Yes, especially when I go swimming.

I: Why?

R: Because swimming requires wearing a swimsuit, which exposes a lot of the body. Sometimes I worry that people may pay attention to my appearance or body shape.

I: Do you think current sports products and services adequately meet women’s needs?

R: Things have improved compared with the past, but there is still room for improvement.

I: In what ways?

R: Many products remain relatively homogeneous, and prices are often quite high. For students, these costs can still be a burden.

I: From a policy perspective, what are your thoughts?

R: I feel that support for women’s sports participation and sports consumption is still quite limited.

R: “I feel that there are still very few policies that specifically support women’s participation in sports or sports consumption.”

R: For example, there are relatively few women-oriented sports facilities, specialized programs, or financial support measures available.

I: Based on your experiences, what do you think society, families, or businesses could do to better support women’s participation in sports?

R: First, we need to reduce gender stereotypes and stop assuming that certain sports are only suitable for men. Second, I hope there will be more affordable sports courses and services. Finally, society should pay greater attention to women’s sports-related needs.

I: Is there anything else you would like to add?

R: Exercise has become a very important part of my life. It is not only about losing weight or improving appearance; it is also a way to become healthier, more confident, and more capable.

I: Those are all my questions. Thank you very much for participating in this interview.

R: Thank you. I hope my experiences can contribute to your research.
