# Supplementary material for: Exploring the motivation and structural barriers of women’s sports consumption from the perspective of the “She-Economy”
Source: PLoS One. 2026 Oct 21;21(10):e0359201. doi: 10.1371/journal.pone.0359201 (PMC13686482; doi:10.1371/journal.pone.0359201)
Supplement: Supplementary file 4 [file pone.0359201.s001.zip › Interview Transcript 5.docx]

Interview Transcript 5

Appendix I: Participant Information Sheet

(Used for participant recruitment and screening prior to the interview and for describing the sample composition in the study)

Interview ID: F5 (To protect privacy, participants are identified by codes such as F01, F02, etc.)

Interview Method:

☑ Online Telephone Interview

□ Face-to-Face Interview

I. Demographic Information

Age: 26

Occupation: Student

Education Level:

□ High School or Below

□ Associate Degree

□ Bachelor's Degree

☑ Master's Degree or Above

Average Monthly Income (Optional):

□ Below RMB 3,000

□ RMB 3,001–6,000

□ RMB 6,001–10,000

□ RMB 10,001–15,000

□ RMB 15,001–20,000

□ Above RMB 20,000

II. Sports Consumption Characteristics

6. What are the one or two sports you participate in most frequently?

Strength Training

7. Approximately how much do you spend on sports-related activities each month? (Including equipment, courses, memberships, sports events, etc.)

□ Below RMB 200

□ RMB 201–500

☑ RMB 501–1,000

□ RMB 1,001–2,000

□ Above RMB 2,000

8. What are your main types of sports consumption? (Multiple choices allowed)

☑ Sports Equipment (clothing, shoes, gear)

☑ Gym/Studio Membership

☑ Personal Training

□ Group Classes (Yoga, Pilates, Aerobics, etc.)

□ Sports Event Tickets

□ Online Fitness App Membership

□ Other: __________

Appendix II: Interview Content

Interviewer (I): Hello, thank you for participating in this academic interview. My name is Shiyi Xuan, and I am a postgraduate student in Sports Humanities and Sociology. I am conducting a research project on women’s sports consumption. The purpose of this interview is to understand your real experiences, thoughts, and feelings regarding sports consumption as a woman. All information will remain confidential and will only be used for academic research. Any information included in future publications will be anonymized. The interview is expected to last about five to six minutes. If you feel uncomfortable with any question, you may skip it or terminate the interview at any time. To ensure accuracy, I would like to record our conversation. Do I have your permission?

Respondent (R): Yes, I agree.

I: Great. Let’s begin. When did you start participating regularly in sports or fitness activities?

R: I started at the beginning of 2024.

I: So your regular exercise habit only began last year. What does your current exercise routine look like? Could you tell me about the frequency, activities, and places where you exercise?

R: I usually go to the gym four or five times a week.

I: That’s quite frequent. What kind of activities do you mainly do there?

R: Mostly strength training.

I: So you mainly train in the weightlifting area? You do not usually work out at home?

R: No, I almost always go to the gym.

I: What are your main sports-related expenditures?

R: I mainly attend a women-only gym and have also purchased personal training sessions.

I: Besides training courses, do you also spend money on sportswear or equipment?

R: Yes. I buy things such as athletic shoes and workout clothes.

I: What was the most recent sports-related purchase that you felt was particularly worthwhile?

R: Probably fitness equipment or accessories such as workout gloves. They last a long time and offer good value for money.

I: What motivates you to continue investing time and money in sports? Is it for health, appearance, stress relief, or something else?

R: Mainly stress relief.Exercise helps stabilize my mood a lot and also improves my sleep.

I: What role does sports play in your social life? Have you ever made purchases because of friends, communities, or fitness influencers?

R: Exercise helps me stay healthier, but my motivation mainly comes from myself rather than other people.

I: Besides physical changes, what psychological or emotional benefits have you gained from exercising?

R: Exercise stimulates dopamine, so I always feel refreshed and in a much better mood after working out.

I: When choosing sports products or services, what factors matter most to you? For example, brand, design, functionality, coaches, or atmosphere?

R: If I’m choosing a gym, I care about whether the facility is spacious and whether the environment is pleasant. If I’m buying equipment or clothing, I focus on functionality as well as appearance.

I: Have you encountered any time-related or financial difficulties while maintaining your sports consumption? How have you dealt with them?

R: Not really. I have sufficient time, and financially I only spend within my means, so there have not been any major difficulties.But,Some people feel that strength training isn’t really suitable for girls.Sometimes, these kinds of views do affect me.And Public sports facilities are not safe enough, so I avoid going at night.

I: What is your family’s attitude toward your sports participation and sports consumption?

R: They are very supportive. However, I sometimes feel uncomfortable when there are too many people in the gym, especially when there are many men around. That is one reason why I eventually chose a women-only gym.

I: Do you feel that certain exercise areas, such as free-weight zones, are designed more for men?

R: Yes. Relatively speaking, there are fewer facilities and designs that specifically consider women’s needs.

I: Do you worry about being judged for your body shape or exercise performance?

R: Yes. When I feel that my movements are not performed correctly, I become a little worried and less confident.

I: Do you think the current market provides sports products and services that adequately meet women’s needs?

R: Not yet.

I: Are there products or services that you feel do not fully meet your expectations?

R: Yes. I feel that my needs are still not completely satisfied by the products currently available.

I: Looking back on your experiences, what do you think society, families, or businesses could do to better support women in enjoying sports?

R: They could pay more attention to women’s needs. For example, dumbbells could be available in a wider range of colors and designs that appeal more to women.

I: Is there anything else you would like to share, or anything that I have not asked but that you think is important for understanding women’s sports consumption?

R: No, that’s all.

I: Thank you very much for taking the time to share your experiences and thoughts. Your insights are extremely valuable to my research. I wish you all the best.

R: Thank you.
