# Supplementary material for: Exploring the motivation and structural barriers of women’s sports consumption from the perspective of the “She-Economy”
Source: PLoS One. 2026 Oct 21;21(10):e0359201. doi: 10.1371/journal.pone.0359201 (PMC13686482; doi:10.1371/journal.pone.0359201)
Supplement: Supplementary file 4 [file pone.0359201.s001.zip › Interview Transcript 6.docx]

Interview Transcript 6

Appendix I: Participant Information Sheet

(Used for participant recruitment and screening prior to the interview and for describing the sample composition in the study)

Interview ID: F6 (To protect privacy, participants are identified by codes such as F01, F02, etc.)

Interview Method:

□ Online Video Conference

☑ Face-to-Face Interview

I. Demographic Information

Age: 24

Occupation: Beverage Specialist

Education Level:

□ High School or Below

□ Associate Degree

☑ Bachelor's Degree

□ Master's Degree or Above

Average Monthly Income (Optional):

□ Below RMB 3,000

☑ RMB 3,001–6,000

□ RMB 6,001–10,000

□ RMB 10,001–15,000

□ RMB 15,001–20,000

□ Above RMB 20,000

II. Sports Consumption Characteristics

6. What are the one or two sports you participate in most frequently?

Night Running, Badminton

7. Approximately how much do you spend on sports-related activities each month? (Including equipment, courses, memberships, sports events, etc.)

□ Below RMB 200

☑ RMB 201–500

□ RMB 501–1,000

□ RMB 1,001–2,000

□ Above RMB 2,000

8. What are your main types of sports consumption? (Multiple choices allowed)

☑ Sports Equipment (clothing, shoes, gear)

□ Gym/Studio Membership

□ Personal Training

□ Group Classes (Yoga, Pilates, Aerobics, etc.)

□ Sports Event Tickets

□ Online Fitness App Membership

□ Other: __________

Appendix II: Interview Content

Interviewer (I): Hello, thank you for participating in this academic interview. My name is Shiyi Xuan, and I am a postgraduate student in Sports Humanities and Sociology. I am conducting a research project on women’s sports consumption. The purpose of this interview is to understand your real experiences, thoughts, and feelings regarding sports consumption as a woman. All information will remain confidential and will only be used for academic research. Any information included in future publications will be anonymized. The interview is expected to last about five to ten minutes. If you feel uncomfortable with any question, you may skip it or terminate the interview at any time. To ensure accuracy, I would like to record our conversation. Do I have your permission?

Respondent (R): Yes.

I: Great. Let’s begin. When did you start participating regularly in sports or fitness activities?

R: I started during my freshman year at university.

I: So you have continued exercising since college, although there may have been some interruptions?

R: Yes, there were some breaks in between.

I: What does your current exercise routine look like? Could you tell me about the frequency, activities, and places where you exercise?

R: My current routine mainly consists of running at night and playing badminton on weekends. Those are my two primary forms of exercise.

I: So night running and badminton are your main activities?

R: Yes.

I: What are your main sports-related expenditures?

R: Mainly sports shoes, badminton shuttlecocks, and badminton rackets.

I: Sports equipment, then?

R: Yes.

I: What was the most recent sports-related purchase that you felt was particularly worthwhile?

R: A badminton racket that I bought recently, and also a pair of running shoes.

I: What motivates you to continue investing time and money in sports? Is it for health, appearance, stress relief, or something else?

R: I think it’s all of those reasons. Exercise benefits my health, helps shape my body, and also allows me to relieve stress from work.Sports are the way I express my attitude toward life.

I: What role does sports play in your social life? Have you ever made purchases because of friends, communities, or influencers?

R: Sports are like a positive addition to my life. I think sportswear, shoes, and equipment have a strong influence on my sports consumption.

I: Have any bloggers or athletes influenced your purchases?

R: Yes.

R: “I saw Yang Shuyu endorsing a sports brand. I thought the shoes she wore looked very comfortable, so I bought the same pair.”

I: Besides physical changes, what psychological or emotional benefits have you gained from exercising?

R: Exercise not only makes my body feel healthier, but it also leaves me feeling refreshed and relaxed afterward. It has had a very positive impact on my mental well-being.

I: When purchasing sports products or services, what factors matter most to you?

R: Comfort is the most important factor. I pay particular attention to comfort and the overall user experience.

I: Have you encountered any time-related or financial difficulties while maintaining sports consumption?

R: Yes.

R: “I’ve seen cases where the same sports shoes cost more just because the color or target group was different—it felt like a pink tax, and it bothered me.”

I: So the issue of the pink tax has caused some frustration for you?

R: Yes, definitely.I think there are no sports programs specifically designed to support women's needs.

I: What is your family’s attitude toward your sports participation and sports consumption?

R: My family is very supportive. They think I do not exercise enough in daily life, so they believe sports are beneficial for my health.

I: You are currently unmarried, correct? So family responsibilities such as childcare have not affected your exercise plans?

R: That’s right. I’m currently unmarried.

I: When you first entered a gym environment, did you ever feel uncomfortable?

R: Yes. When I first went to a gym, I felt somewhat uncomfortable. The atmosphere, people’s gazes, and concerns about appearance sometimes made me uneasy.

I: Do you feel that certain exercise areas, such as free-weight zones, are designed more for men?

R: To some extent, yes. Although it may partly reflect stereotypes, I do occasionally feel that way.

I: Do you worry about people judging your body shape or exercise performance?

R: A little. However, if someone corrects my exercise posture or technique, I would actually appreciate the feedback.

I: Do you think the current market provides sports products and services that adequately meet women’s needs?

R: Not entirely.

I: Are there products or services that you feel do not fully meet your expectations?

R: Yes. For example, there are more male coaches in gyms. As a woman, I would sometimes prefer guidance from a female coach.

I: Looking back on your experiences, what do you think society, families, or businesses could do to better support women in enjoying sports?

R: I think society could do more to promote the benefits of exercise for women and highlight women’s enthusiasm for sports and fitness. That might encourage more women to participate in physical activity.

I: Is there anything else you would like to share, or anything that I have not asked but that you think is important for understanding women’s sports consumption?

R: No, nothing else at the moment.

I: Thank you very much for taking the time to share your experiences and thoughts. Your insights are extremely valuable to my research. I wish you all the best.

R: Thank you.
