# Supplementary material for: Exploring the motivation and structural barriers of women’s sports consumption from the perspective of the “She-Economy”
Source: PLoS One. 2026 Oct 21;21(10):e0359201. doi: 10.1371/journal.pone.0359201 (PMC13686482; doi:10.1371/journal.pone.0359201)
Supplement: Supplementary file 4 [file pone.0359201.s001.zip › Interview Transcript 7.docx]

Interview Transcript 7

Appendix I: Participant Information Sheet

(Used for participant recruitment and screening before the interview and for describing sample characteristics in the study)

Interview ID: F7 (Pseudonym used to protect participant privacy)

Interview Method:

□ Online Telephone Interview

☑ Face-to-Face Interview

I. Demographic Information

Age: 27

Occupation: Master's Student

Education Level:

□ High School or Below

☑ Associate Degree

□ Bachelor's Degree

☑ Master's Degree or Above

Average Monthly Income (Optional):

□ Below RMB 3,000

☑ RMB 3,001–6,000

□ RMB 6,001–10,000

□ RMB 10,001–15,000

□ RMB 15,001–20,000

□ Above RMB 20,000

II. Sports Consumption Characteristics

6. What are the 1–2 sports you participate in most frequently?

Marathon Running

7. Approximately how much do you spend on sports-related activities each month? (Including equipment, courses, memberships, event tickets, etc.)

□ Below RMB 200

☑ RMB 201–500

□ RMB 501–1,000

□ RMB 1,001–2,000

□ Above RMB 2,000

8. What are your primary forms of sports consumption? (Multiple choices allowed)

☑ Sports equipment (clothing, shoes, gear)

☑ Gym/studio membership

□ Personal training

□ Group classes (yoga, Pilates, aerobics, etc.)

□ Sports event tickets

□ Fitness app memberships

□ Other: __________

Appendix II: Interview Transcript

Interviewer (I): Hello! Thank you for participating in this academic interview. My name is Shiyi Xuan Shi, and I am a postgraduate student in Sport Humanities and Sociology. I am conducting a research project on women’s sports consumption.

This interview aims to understand your real experiences, feelings, and perspectives regarding sports consumption as a woman. All information will be kept strictly confidential and used only for academic research purposes. All personal information will be anonymized in the final thesis.

The interview will take approximately five to six minutes. If any question makes you uncomfortable, you may skip it or terminate the interview at any time. To ensure accuracy, I would like to record the interview. Do you agree?

Respondent (R): Yes, that's fine.

I: When did you begin participating in sports regularly?

R: I started running consistently during my undergraduate years. At first, I mainly wanted to improve my fitness and control my weight. Over time, I gradually developed a passion for long-distance running. After entering graduate school, running became a very important part of my life, and I also started participating in marathon races.

I: What does your current exercise routine look like?

R: My main activities are marathon running and regular running training. I usually run four to five times a week, with each session lasting about an hour. On weekends, I typically schedule long-distance training runs. I usually run on the university track, around campus, or in city parks. If I am preparing for a race, I follow a structured training plan.

I: What are your main sports-related expenditures?

R: Mainly running shoes, sportswear, sports watches, and marathon registration fees. Sometimes participating in races outside my city also involves transportation and accommodation expenses. One of the most worthwhile purchases I've made recently was a pair of professional marathon running shoes. They significantly improved my running experience and reduced discomfort during training.

I: What motivates you to continue investing time and money in sports?

R: Initially, it was probably for weight loss and maintaining my figure. But after sticking with it for years, health is no longer the only reason. Running has become more of a lifestyle for me. Graduate school comes with considerable academic pressure, and running helps me relieve stress and maintain emotional stability.

I: What role does sports play in your social life?

R: It plays a fairly important role. I’ve met many fellow runners and joined several running communities. We exchange training experiences, equipment recommendations, and race information. Sometimes I purchase products because of recommendations from other runners rather than advertisements.

I: Besides physical changes, what psychological or emotional benefits has sports brought you?

R: “Besides the changes in my weight, sports make me feel a stronger sense of self and improve my quality of life.”

I: Could you elaborate on that?

R: “I’ve been running marathons for a long time. Beyond the obvious physical changes, it’s really the sense of accomplishment I get from pushing myself over and over again that satisfies me mentally.”

Every time I complete a race or achieve a personal best, I feel an incredible sense of achievement.

I: How has that feeling influenced your life?

R: It has made me more confident. Marathon running is a constant process of challenging yourself. It teaches perseverance, self-discipline, and how to face difficulties. These qualities also influence other aspects of my academic and personal life. That is why I believe sports bring much more than physical benefits.

I: What do you value most when selecting sports products or services?

R: Functionality is definitely the top priority. When purchasing running shoes, I focus most on comfort, support, and protection rather than color or appearance. For sports services, I care more about professionalism and whether the service genuinely meets my needs.

I: Have you encountered any time or financial difficulties while maintaining sports consumption?

R: Time is probably the bigger challenge. Graduate studies involve heavy research tasks, including reading literature and writing papers, so I need to carefully schedule my training sessions. Financially, race registration fees, transportation, accommodation, and equipment upgrades can also create pressure. However, I still believe these investments are worthwhile.

I: What is your family’s attitude toward your participation in sports and sports consumption?

R: My family is generally very supportive because they believe exercise is beneficial for health. Since I am currently unmarried and do not have children, I have more flexibility in managing my time compared with many married women.

I: Have you ever felt uncomfortable or embarrassed in public sports spaces?

R: Yes. Particularly in the free-weight area of gyms, where there are usually more men. Sometimes I worry whether my movements are correct or feel that people might be paying attention to me, which can make me feel somewhat uncomfortable.

I: Do you think certain sports spaces are designed more for men?

R: To some extent, yes. The atmosphere, equipment layout, and user composition in strength-training areas often create a feeling that these spaces primarily belong to men. No one explicitly says so, but the environment can communicate that message.

I: Do you worry about being judged for your body shape or athletic performance?

R: Occasionally. Women often seem to be more affected by body-related evaluations. Even when I am simply exercising, I sometimes worry that others might judge my appearance, movements, or performance.

I: Do you think current sports products and services adequately meet women's needs?

R: I think there is still room for improvement. Some so-called women-specific products simply change the color or appearance without genuinely addressing women's actual needs. I hope companies can design products based on women’s exercise habits and physical characteristics.

I: Based on your experiences, what could society, families, or companies do to better support women’s participation in sports?

R: I think the most important thing is creating a more inclusive environment. Society should encourage women to exercise for health, enjoyment, and personal growth, rather than simply associating sports with weight loss or beauty standards. Family members can provide greater understanding and support, while companies should pay more attention to women’s genuine sports needs instead of relying on stereotypes.

I: Is there anything else you would like to add?

R: I believe sports are not merely a form of consumption for women; they are also a lifestyle and a process of self-development. In many cases, the confidence and sense of empowerment gained through sports are far more important than physical changes. I hope more women can find sports that suit them and truly enjoy the happiness that exercise brings.

I: Those are all my questions. Thank you very much for sharing your genuine and valuable experiences.

R: Thank you. I hope my experiences can contribute to your research.
