# Supplementary material for: Exploring the motivation and structural barriers of women’s sports consumption from the perspective of the “She-Economy”
Source: PLoS One. 2026 Oct 21;21(10):e0359201. doi: 10.1371/journal.pone.0359201 (PMC13686482; doi:10.1371/journal.pone.0359201)
Supplement: Supplementary file 4 [file pone.0359201.s001.zip › Interview Transcript 8.docx]

Interview Transcript 8

Appendix I: Participant Information Sheet

(Used for participant recruitment, screening, and sample description in the study)

Interview ID: F8

Interview Method:

□ Online Telephone

☑ Face-to-Face

I. Demographic Information

Age: 30

Occupation: University Staff Member

Education Level:

□ High School or Below

□ Associate Degree

□ Bachelor's Degree

☑ Master's Degree or Above

Average Monthly Income（Optional）:

□ Below RMB 3,000

□ RMB 3,001–6,000

☑ RMB 6,001–10,000

□ RMB 10,001–15,000

□ RMB 15,001–20,000

□ Above RMB 20,000

II. Sports Consumption Characteristics

6. What sports do you participate in most frequently?

Fitness Training / Strength Training

7. Approximately how much do you spend on sports-related activities each month?

□ Below RMB 200

□ RMB 201–500

☑ RMB 501–1,000

□ RMB 1,001–2,000

□ Above RMB 2,000

8. What types of sports consumption do you mainly engage in? (Multiple choices allowed)

☑ Sports Equipment (sportswear, shoes, gear)

☑ Gym/Studio Membership

☑ Group Fitness Classes (Yoga, Pilates, Aerobics, etc.)

☑ Online Fitness App Membership

□ Personal Training

□ Sports Event Tickets

□ Other: ________

Appendix II: Interview Content

Interviewer (I): Hello, thank you for participating in this academic interview. My name is Shiyi Xuan, and I am a postgraduate student majoring in Sports Humanities and Sociology. I am conducting research on women's sports consumption. The purpose of this interview is to understand your experiences, perceptions, and thoughts regarding sports consumption as a woman. All information will remain confidential and will be used solely for academic purposes. To ensure accuracy, I would like to record the interview. Do you agree?

Respondent (R): Yes, that's fine.

I: When did you begin participating in sports regularly?

R: I started exercising regularly after I began working. At that time, my job involved a lot of sitting, and I often felt physically tired and mentally stressed. Some colleagues invited me to try fitness classes, and I gradually developed the habit of going to the gym.

I: What does your current exercise routine look like?

R: I usually go to the gym three or four times a week, and each session lasts around one and a half hours. Most of my workouts involve strength training and cardio exercises. Occasionally, I also attend yoga or Pilates classes. I usually work out after work on weekdays and spend more time exercising on weekends.

I: What are your main sports-related expenditures?

R: My major expenses include gym membership fees, sportswear, training shoes, and online fitness courses. Recently, the most worthwhile purchase for me was a set of professional workout clothes. They are comfortable and significantly improved my workout experience.

I: What motivates you to continuously invest time and money in sports?

R: Initially, it was mainly for weight control and improving my appearance. However, over time I realized that exercise has become an important way to relieve stress and maintain emotional well-being. It helps me feel more balanced and in control of my life.

I: What role does sports participation play in your social life?

R: It plays quite an important role. I have several friends who regularly work out with me. We often attend classes together and motivate each other. I also follow several fitness influencers on social media.

I: Do these influencers affect your consumption decisions?

R: Yes, they do. Sometimes their workout content or product recommendations attract my attention. In fact, one sentence perfectly describes my experience: “Sometimes when I see a fitness vlogger I follow posting a new workout video, I can’t help wanting to move my body too.”

I: Besides physical changes, what psychological or emotional benefits have you gained from exercise?

R: The biggest change is increased confidence. Before I started exercising regularly, I was often anxious and dissatisfied with my body. Exercise has made me feel more positive, energetic, and confident.

I: What factors do you value most when choosing sports products or services?

R: Functionality is the most important factor, followed by comfort. For fitness classes, I pay attention to the professionalism of the instructors and whether the environment feels welcoming. Price matters, but value for money is more important.

I: Have you experienced any time or financial difficulties in maintaining sports consumption?

R: Time is definitely the biggest challenge. Although university jobs are relatively stable, there are many administrative tasks and unexpected responsibilities. Sometimes I finish work very late and have to decide whether I still have enough energy to go to the gym.

I: How do your family members or spouse view your sports participation and sports-related spending?

R: My husband is generally supportive. However, I sometimes feel psychological pressure myself because traditional gender expectations still influence me.

I: Could you explain that further?

R: Sometimes after work, when I go to the gym with friends or shop for sports gear instead of going home to cook or help my kid with homework, I always feel a bit guilty.

R: To be honest, I often have thoughts like this: “Sometimes after work, when I go to the gym with friends or buy some sports gear instead of going home to cook or help my kid with homework, I feel really uneasy.” Even though I do not currently have children, I still feel the influence of social expectations regarding women's family responsibilities.

I: Have you ever felt uncomfortable or embarrassed in public sports spaces?

R: Yes, quite often.

I: Could you give an example?

R: I have always wanted to learn swimming, but I never actually tried.

I: Why not?

R: Mainly because of body image concerns. As I often say, “I always wanted to learn swimming, but I never tried because swimsuits show a lot of skin, and swimming pools are crowded. My body doesn’t look good, so I felt uncomfortable going.”

I: Do you worry about being judged based on your body shape or athletic performance?

R: Yes. I think many women have experienced that. Gyms are often filled with mirrors, and sometimes I become overly aware of whether others are looking at me. Occasionally, I even hesitate to try new exercises because I worry about appearing awkward or unskilled.

I: Do you think current sports products and services adequately meet women’s needs?

R: Things have improved compared with the past, but there is still room for improvement. Many products marketed as “female-oriented” focus mainly on colors and appearance rather than women's actual exercise needs. I would like to see more products and services designed based on women's real experiences.

I: Based on your experiences, what could society, families, or businesses do to better support women's participation in sports?

R: First, society should reduce stereotypes about women and stop treating exercise merely as a way to lose weight or improve appearance. Families should provide more understanding and support. Businesses should genuinely understand women consumers rather than relying on superficial assumptions.

I: Is there anything else you would like to add?

R: I believe sports participation is not just a form of consumption; it is also a lifestyle and a process of personal growth. For many women, the biggest barriers are not necessarily money or time, but social judgments and internalized pressure. If these pressures could be reduced, more women would be willing to enjoy sports.

I: Thank you very much for participating in this interview and sharing your valuable experiences.

R: Thank you. I hope my experiences can contribute to your research.
