# Supplementary material for: Exploring the motivation and structural barriers of women’s sports consumption from the perspective of the “She-Economy”
Source: PLoS One. 2026 Oct 21;21(10):e0359201. doi: 10.1371/journal.pone.0359201 (PMC13686482; doi:10.1371/journal.pone.0359201)
Supplement: Supplementary file 4 [file pone.0359201.s001.zip › Interview Transcript 9.docx]

Interview Transcript 9

Appendix I: Participant Information Sheet

(Used for participant recruitment and screening before the interview and for describing sample characteristics in the study)

Interview ID: F9 (Pseudonym used to protect participant privacy)

Interview Method: ☑ Face-to-face Interview □ Online Video Conference

I. Demographic Information

Age: 24

Occupation: Teacher at a Private Educational Training Institution

Education Level:

□ High School or Below

□ Associate Degree

☑ Bachelor's Degree

□ Master's Degree or Above

Average Monthly Income (Optional):

□ Below RMB 3,000

☑ RMB 3,001–6,000

□ RMB 6,001–10,000

□ RMB 10,001–15,000

□ RMB 15,001–20,000

□ Above RMB 20,000

II. Sports Consumption Characteristics

6. What are the 1–2 sports you participate in most frequently?

Badminton

7. Approximately how much do you spend on sports-related activities each month? (Including equipment, courses, memberships, event tickets, etc.)

☑ Below RMB 200

□ RMB 201–500

□ RMB 501–1,000

□ RMB 1,001–2,000

□ Above RMB 2,000

8. What are your primary forms of sports consumption? (Multiple choices allowed)

☑ Sports equipment (clothing, shoes, gear)

□ Gym/studio membership

□ Personal training

□ Group classes (yoga, Pilates, aerobics, etc.)

□ Sports event tickets

□ Fitness app memberships

□ Other: __________

Appendix II: Interview Transcript

Interviewer (I): Hello, thank you for participating in this academic interview. My name is Shiyi Xuan Shi, and I am a postgraduate student in Sport Humanities and Sociology. I am conducting a study on women’s sports consumption. This interview aims to understand your real experiences, thoughts, and feelings regarding sports consumption as a woman. All information will be kept confidential and used solely for academic research purposes. All personal information will be anonymized in the final thesis. The interview is expected to last about 20 minutes. If you feel uncomfortable with any question, you may skip it or stop the interview at any time. To ensure accuracy, I would like to record the interview. Do you agree?

Participant (O): Yes, I agree.

I: Great. To begin, could you tell me when you started participating in sports or fitness activities regularly?

O: I started after graduating from high school and entering university because I had a lot of free time.

I: What are your current exercise habits in terms of frequency, activities, and locations?

O: After graduation, I usually go to a park in the morning to play badminton or take a walk.

I: What are your main sports-related expenditures?

O: Mainly badminton rackets and shuttlecocks.

I: Shuttlecocks have become quite expensive recently, haven't they?

O: Yes.

O: “Badminton and the equipment—like rackets and accessories—are getting more and more expensive now.”

I: What was the most worthwhile sports-related expense you have made recently?

O: A newly opened sports center near my area. The environment is much better than outdoor courts or smaller venues, and the playing experience is much better.

I: What motivates you to continue investing time and money in sports?

O: Exercise makes me feel energetic. If I spend the whole day sitting and working or studying, I feel bored. Once I exercise, I immediately feel happier and more energized.

I: Have your sports consumption decisions ever been influenced by friends, communities, celebrities, or athletes?

O: Rarely. I usually only spend money on things that I personally like. Celebrity endorsements seldom influence me.

I: Besides emotional benefits, have you experienced any physical changes through exercise?

O: Yes. I used to weigh around 120 jin (about 60 kg). After I started playing badminton regularly, I became less focused on eating and more focused on exercise. Now I weigh around 110 jin.

I: When selecting sports products or services, what factors matter most to you?

O: I care most about functionality and appearance. If a product has a particularly attractive design, it catches my attention.

I: Have you encountered time or financial difficulties while maintaining sports consumption?

O: Sometimes work keeps me busy, so I postpone exercise until weekends or after work. When the commute takes too much time, so I have to rely on online fitness.Financially, if sports-related costs increase, I simply reduce purchases and continue using existing equipment.

I: So rising costs can create some financial pressure?

O: Yes. If badminton becomes too expensive, I may adjust how often I play or switch to different equipment, but I would never stop exercising altogether.

I: Have you ever felt uncomfortable or embarrassed in public sports spaces such as gyms or parks?

O: Yes.

O: “One reason I chose badminton was that in many gyms around here, most people are men, and I don’t feel very comfortable.”

O: Also, when I go alone, I sometimes worry that my movements are not professional enough, so I feel a little embarrassed.

I: Do you think certain sports spaces are designed more for men?

O: To some extent, yes.

O: “When we play badminton, the surrounding ping-pong and basketball courts are mostly occupied by men. There are very few women. Most areas are still male-dominated, so it feels a bit embarrassing to go there.”

I: Do you worry about being judged for your body shape or exercise performance?

O: If people comment on my movements, I can accept it because it may help me improve. However, I do not like comments about my body shape.

I: Do you think current sports products and services adequately meet women’s needs?

O: Generally speaking, yes. There are already many products designed specifically for women. Many of them are aesthetically pleasing, colorful, and attractive.

I: Do you think the so-called “pink tax” causes inconvenience for you?

O: I can accept it to some extent. If a product is especially beautiful or appealing, I am willing to pay for its appearance.

I: Is there anything else you would like to share that you think is important for understanding women’s sports consumption?

O: No, not really.

I: Thank you very much. Those are all my questions. I sincerely appreciate you taking the time to share your experiences and opinions. Your insights are extremely valuable to my research. I wish you all the best.

O: Thank you.
